# Metabolic and Immunological Subtypes of Esophageal Cancer Reveal Potential Therapeutic Opportunities

**DOI:** 10.3389/fcell.2021.667852

**Published:** 2021-07-08

**Authors:** Ryan J. King, Fang Qiu, Fang Yu, Pankaj K. Singh

**Affiliations:** ^1^The Eppley Institute for Research in Cancer and Allied Diseases, University of Nebraska Medical Center, Omaha, NE, United States; ^2^Department of Biostatistics, University of Nebraska Medical Center, Omaha, NE, United States; ^3^Department of Biochemistry and Molecular Biology, University of Nebraska Medical Center, Omaha, NE, United States; ^4^Department of Genetics, Cell Biology and Anatomy, University of Nebraska Medical Center, Omaha, NE, United States; ^5^Department of Pathology and Microbiology, University of Nebraska Medical Center, Omaha, NE, United States; ^6^Fred & Pamela Buffett Cancer Center, University of Nebraska Medical Center, Omaha, NE, United States

**Keywords:** metabolic subtypes, immunological subtypes, esophageal cancer, cancer metabolism, biomarkers

## Abstract

**Background:**

Esophageal cancer has the sixth highest rate of cancer-associated deaths worldwide, with many patients displaying metastases and chemotherapy resistance. We sought to find subtypes to see if precision medicine could play a role in finding new potential targets and predicting responses to therapy. Since metabolism not only drives cancers but also serves as a readout, metabolism was examined as a key reporter for differences.

**Methods:**

Unsupervised and supervised classification methods, including hierarchical clustering, partial least squares discriminant analysis, k-nearest neighbors, and machine learning techniques, were used to discover and display two major subgroups. Genes, pathways, gene ontologies, survival, and immune differences between the groups were further examined, along with biomarkers between the groups and against normal tissue.

**Results:**

Esophageal cancer had two major unique metabolic profiles observed between the histological subtypes esophageal squamous cell carcinoma (ESCC) and esophageal adenocarcinoma (EAC). The metabolic differences suggest that ESCC depends on glycolysis, whereas EAC relies more on oxidative metabolism, catabolism of glycolipids, the tricarboxylic acid (TCA) cycle, and the electron transport chain. We also noted a robust prognostic risk associated with *COQ3* expression. In addition to the metabolic alterations, we noted significant alterations in key pathways regulating immunity, including alterations in cytokines and predicted immune infiltration. ESCC appears to have increased signature associated with dendritic cells, Th17, and CD8 T cells, the latter of which correlate with survival in ESCC. We bioinformatically observed that ESCC may be more responsive to checkpoint inhibitor therapy than EAC and postulate targets to enhance therapy further. Lastly, we highlight correlations between differentially expressed enzymes and the potential immune status.

**Conclusion:**

Overall, these results highlight the extreme differences observed between the histological subtypes and may lead to novel biomarkers, therapeutic strategies, and differences in therapeutic response for targeting each esophageal cancer subtype.

## Introduction

Precision medicine holds strong potential for delivering more promising therapeutics to the correct patients. One subset of patients may respond strongly to a given therapy, while another subset may show little to no improvement, thereby increasing the risk of futilely decreasing quality of life for patients. Differences in cancer etiology, environments, and evolutionary selection *via* selection pressures from founding mutations may give rise to different subsets of cancer profiles, which can have different vulnerabilities and inherent resistances.

Metabolism is a key reporter of cellular status. It is no surprise that founding mutations of cancers alter metabolism. Either all functional genes utilize a metabolic reaction to exert their function or their alteration impacts the cell in a manner that affects proteins utilizing a metabolic reaction. It has been observed that expression of enzyme-coding messenger RNAs (mRNAs) can be utilized to estimate metabolic flux (Lee et al., 2012; Mehrmohamadi et al., 2014; Xiao et al., 2019). This study hypothesizes that metabolic alterations can reveal unique cancer subtypes that can give rise to precision medicine based on differences in clinical attributes and their associated selection that results from environmental factors and cancer etiology. Hence, this study seeks to utilize metabolic enzyme expression as a reporter to determine if differences can give rise to distinctive subtypes that are uniquely targetable. The following 13 cancer cohorts were screened through The Cancer Genome Atlas (TCGA): esophageal carcinoma, kidney clear cell renal carcinoma (KIRC), kidney renal papillary carcinoma, lung adenocarcinoma, lung squamous cell carcinoma, prostate adenocarcinoma acinar type, pancreatic adenocarcinoma, colon adenocarcinoma, rectum adenocarcinoma, bladder urothelial carcinoma, breast invasive carcinoma, stomach adenocarcinoma, and uterine corpus endometrial carcinoma. Of these 13 cohorts, only esophageal cancer showed a drastic change when patients were separated based on attribute of histological subtype.

Worldwide, esophageal cancer is the eighth most common cancer, presenting with the sixth highest rate of cancer-associated deaths (Pisani et al., 1999; Ferlay et al., 2010). The 5-year overall survival rate in the United States is 19.9% (Howlader et al., 2019), and the rate of incidence and associated mortality has increased 15–20% in the last 30 years (Enzinger and Mayer, 2003). Although esophageal squamous cell carcinoma (ESCC) is more prevalent than esophageal adenocarcinoma (EAC) worldwide (Enzinger and Mayer, 2003; Spechler et al., 2006), the diagnosis rate of EAC has also increased by over 600% in the last 30 years in the United States alone (Spechler et al., 2006). Differences in lifestyle and associated etiological factors may give rise to these different subtypes of esophageal cancer (Enzinger and Mayer, 2003), which could be targeted more precisely based on the subtype.

Roughly half of the patients with esophageal cancer present with distant metastases and are treated with chemotherapies despite heterogeneity-associated resistance of ESCC and EAC to chemotherapy (Huang and Fu, 2019; Zhao et al., 2019). These observations have prompted the field to examine potential immunological approaches for treatment (Huang and Fu, 2019; Kelly, 2019; Zhao et al., 2019). Due to differences in metabolism between subtypes and the extent to which metabolism can exert influence over the tumor microenvironment (Chang et al., 2015; Allard et al., 2017; Gupta et al., 2017; Rivadeneira and Delgoffe, 2018; Sugiura and Rathmell, 2018; Triplett et al., 2018; Chen et al., 2019; Najjar et al., 2019; Ngwa et al., 2019; Thapa and Lee, 2019; Vigano et al., 2019), this study examined subtypes based on the extent of metabolic differences, clinical parameters, and the cytokine environment. This study also examined their potential roles in immunity and checkpoint therapy. Overall, the study suggests that potential metabolic and immune differences in tumor subtypes can be exploited with precision medicine.

## Materials and Methods

### Data Retrieval

The Cancer Genome Atlas data were downloaded using TCGA Data Matrix on October 5, 2015. Esophageal feature selection and machine learning were downloaded in October 2020 through “TCGAbiolinks” v2.16.4 on R 4.0.2 in R studio v1.3.1093. Mapping gene symbols to ensemble gene IDs was done through “ensembldb” v3.11 (Rainer et al., 2019), and duplicated names that were not mRNA were dropped from the analysis. TCGA mRNA and clinical attributes were analyzed as performed previously (King et al., 2017). RNA sequencing by expectation-maximization (RSEM) expression for mRNA was obtained as upper quartile-normalized RSEM for the given TCGA cohort. A list of human metabolic enzymes was downloaded from the Kyoto Encyclopedia of Genes and Genomes (KEGG) pathway for hsa01100. Gene Ontology (GO) and KEGG pathways were from the resources that were supplied with Gene Set Enrichment Analysis (GSEA) v2.2.3. Only pathways that contained ≥90% of the genes coding for enzymes with a minimum of 10 enzymatic genes per pathway, as defined from hsa01100, were considered metabolically associated. The entire GO pathway, including cellular component, molecular function, and biological process (BP), was kept for a global perspective for Figure 3A after being filtered for metabolically associated pathways. The remaining GO pathway analyses use only BP to find functional biological meaning.

### Data and Statistical Analyses

ActiveState Perl5 version 5.24.1^1^ was used to gather and organize data, perform Student’s *t*-tests, Benjamini–Hochberg corrections, and quartile quantifications, to feed commands to GSEA through Java, to generate and execute R scripts, and to record the output. Bar graphs were plotted and analyzed in GraphPad Prism 5 (GraphPad Software Inc., San Diego, CA, United States). Machine learning was conducted solely in R using the R package randomForest and H2O v3.32.0.1 (Liaw and Wiener, 2001; H2O.ai, 2016). Cohorts were randomly assigned with a seed of 123 giving 80% of the data for training in the non-tuned randomForest, while 40% of the data were used for training in H2O. Twenty percent of the data were used for validation and testing in H2O with a maximum of 200 models generated for hyperparameter tuning, when applicable, for distributed random forest, gradient boosting machine, deep learning, and generalized linear model. In all machine learning cases, a seed of 123 was set prior for the run.

Partial least-squares discriminant analysis (PLS-DA) was generated through the R package “mixOmics” (Le Cao et al., 2009; Gonzalez et al., 2012; Rohart et al., 2017). mixOmics v6.12.2 was utilized for feature selection through sparse partial least-squares discriminant analysis (sPLS-DA), subsequent tuning, and the resulting performance assessment. Upper quartile-normalized RSEM was converted to log_2_(RSEM +1) before scaling. All randomization events were preset with a seed of 123. Feature selection tuning grid consisted of evaluating the performance when including 1–10, 20, 30, 40, 50, 60, 70, 80, 90, 100, 110, 120, 130, 140, 150, 160, 170, 180, 190, 200, and 300 genes. For EAC vs. ESCC sPLS-DA discrimination, 80% of the cohorts went into training with rounding in effect after randomization. For EAC vs. ESCC vs. normal tissue, 75% of the data went to training, as 80% resulted in a testing cohort having only two normal adjacent tissue samples. Cross-validation was done with leave-one-out (LOO). The area under the curve (AUC) was analyzed using the R package “pROC” v1.16.2 (Robin et al., 2011).

R versions 3.3.2 and 3.5.1^2^ were responsible for the remaining analysis, including heatmaps through the R package “gplots” (Warnes et al., 2020). For Supplementary Figure 5, hierarchical cluster analysis was performed using Ward’s minimum-variance method and applied to data with greater variability using the “factoextra” package in R, while heatmaps were generated using Genesis 1.8.1 (Graz University of Technology, Graz, Austria). Overall survival in Supplementary Figure 5 was plotted using the Kaplan–Meier method and compared between cluster groups using log-rank tests *via* SAS version 9.4 (SAS Institute, Cary, NC, United States). Survival analyses for the genes in the Supplementary Tables were analyzed with the function “survdiff” from R package “survival” using the Mantel–Haenszel log-rank test (Grambsch and Therneau, 2000; Therneau, 2020). When Kaplan–Meier curves were presented, *p*-values were from GraphPad Prism 5, using the Mantel–Cox log-rank test for significance. The Mann–Whitney *U* test was conducted in R with function “wilcox.test,” and Spearman’s correlations were calculated utilizing R package “Hmisc” (Harrell and Dupont, 2020).

GraphPad Prism 5 was also utilized to calculate Mann–Whitney U or Student’s *t*-test when two categories existed and Kruskal–Wallis *H* test or one-way ANOVA with Bonferroni’s multiple comparison test when more than two categories existed. Error bars represent the standard error of the mean. Prism also calculated Fisher’s exact test when two categorical categories existed, and chi-square was used when there were more than two categorical categories, except where mentioned.

### Immune Infiltration Prediction Algorithms

CIBERSORT (Abbas et al., 2009) was analyzed through the project’s website^3^ utilizing 1,000 permutations with quantile normalization disabled, for the downloaded TCGA data already had upper quartile normalization. The Tumor Immune Dysfunction and Exclusion (TIDE) (Jiang et al., 2018) was run through the project’s main website^4^ utilizing “Other” cancer without previous immunotherapy after supplying log_2_(RSEM+1) with normalization to the average of the entire esophageal cohort. The xCell (Aran et al., 2017) data were generated from the project’s website^5^. The docker provided by tumor immunology miner (TIminer) (Tappeiner et al., 2017) was downloaded from the project’s website^6^, and the.gmx files for Angelova et al. (2015) and Charoentong et al. (2017) were extracted to be run by GSEA utilizing pre-ranked files generated by comparing the relative RSEM expression of each patient to the average of the normal adjacent tissue. Due to similar results for Th17 cells, the signature of Angelova et al. (2015) was used to represent TIminer due to the computation time.

## Results

### Esophageal Squamous Cell Carcinoma and Esophageal Adenocarcinoma Present Unique Metabolic Gene Signatures

**FIGURE 1:**
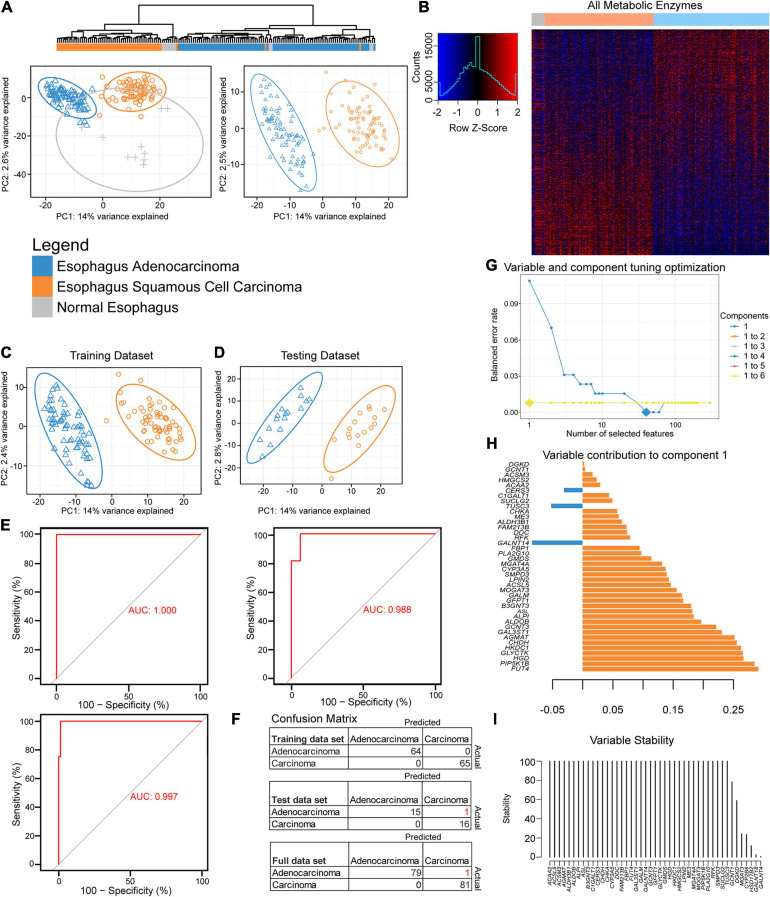
Partial least-squares discriminant analysis (PLS-DA) reveals histological differences for metabolic enzymes. **(A)** Hierarchical and PLS-DA clustering of enzyme mRNA levels between esophageal adenocarcinoma (EAC), esophageal squamous cell carcinoma (ESCC), and normal tissue (left) and just the histological subtypes (right). **(B)** Heatmap row Z-scores of log_2_ mRNA expression. **(C,D)** PLS-DA containing scaled log_2_ enzyme mRNA for 80% of each histological subtype that was randomly sent to training **(C)**, while the remainder went to the testing dataset **(D)**. **(E)** The receiver operating characteristic (ROC) curve of the training (upper left), testing (right), and the full dataset (bottom). **(F)** The confusion matrix with incorrect predicted classifications shown in red after tuning the sparse partial least-squares discriminant analysis (sPLS-DA). **(H)** The optimal combination of one component and 40 enzyme expression was further examined for their eigenvalues (influence) on component 1. **(G)** The balanced error rate for the first six components with 1–10, 20, 30, 40, 50, 60, 70, 80, 90, 100, 110, 120, 130, 140, 150, 160, 170, 180, 190, 200, or 300 enzyme mRNA expression(s) included in the tuned sPLS-DA. Large diamonds indicate the lowest error rate for the given component and number of enzymes. Components 3–6 could not decrease the balanced error rate further and are hidden behind the line of the second component. **(I)** The 40 enzymes in component 1 were examined for stability.

Thirteen different cancer cohorts were downloaded from TCGA to search for metabolic distinctions between clinical attributes, including sex, stage, ethnicity, and histological subtypes. Each of these covariates was controlled, and different permutations were examined for potential differences, as visualized through PLS-DA. When examining histological subtypes, many cancers appeared to have metabolic gene expression differences between adjacent normal tissue and cancerous tissue (Supplementary Figure 1), which is in line with Warburg’s observation and previous publications (Vander Heiden et al., 2009; Chaika et al., 2012). However, while the majority of TCGA cohorts examined appeared not to have any metabolic differences between histological subtypes, slight differences exist in breast, rectal, and uterine cancer subtypes (Supplementary Figure 2). One exception is the astounding metabolic differences observed between ESCC and EAC (Figure 1A). The univariate analysis revealed a global change in metabolic enzyme mRNA expression levels (Figure 1B), confirming the observation of differential metabolic regulation between ESCC and EAC histological subtypes.

A feature selection approach was used through mixOmics (Rohart et al., 2017), utilizing only enzyme mRNA expression to discover the key metabolic differences between the subtypes by tuning an sPLS-DA. Patients were separated by histological subtypes and randomly assigned 80% to the training cohort (Figure 1C) with the remaining assigned to the testing cohort (Figure 1D). The resulting training dataset fit well with a perfect performance, but the testing cohort made one mistake in 32 patients (Figures 1E,F). The responsible enzymes for the tuned sPLS-DA separation were further examined for their contribution (Figure 1G). Combining up to six components was examined; however, utilizing more than the first principal component had a negligible performance impact after the first component, as the first component gave the lowest balanced error rate (Figure 1H). The 40 genes that were comprised within the first component were found to be mostly stable (Figures 1G,I).

We next examined the performance by evaluating if enzyme expression could be used to separate normal adjacent tissue from the two histological cohorts (Supplementary Figure 3A). Seventy-five percent of the data were randomly sent to training (Supplementary Figure 3B) and 25% to testing (Supplementary Figure 3C). The tuning grid showed that two components were optimal (Supplementary Figure 3D). The first component’s top genes were similar to the component for separating EAC from ESCC (Figure 1H and Supplementary Figure 3E) and that component 2 helps in the separation of normal tissue from the rest (Supplementary Figure 3F). While the majority of the genes were mostly stable, there was difficulty in separating normal tissue from the rest with feature selection in the training and testing dataset (Supplementary Figures 3H–J), despite optimistic separation with limited testing sample size in the sPLS-DA (Supplementary Figure 3C). The final sPLS-DA shows a slight cluster of normal tissue, with distant normal adjacent tissue, EAC-like normal tissue, as well as ESCC-like normal tissue. This begs the question if there is minor local influence on adjacent normal tissue’s metabolic phenotype or if the normal tissue was not as pure as desired (Supplementary Figure 3K). To further chase differences, we next examined the entire expression of mRNA-encoding genes and also found good separation (Supplementary Figure 3L). Tuning the sPLS-DA suggested just one component and one gene, *GPR35*, was sufficient to separate these with decent stability and performance (Supplementary Figures 3M–P).

**FIGURE 2:**
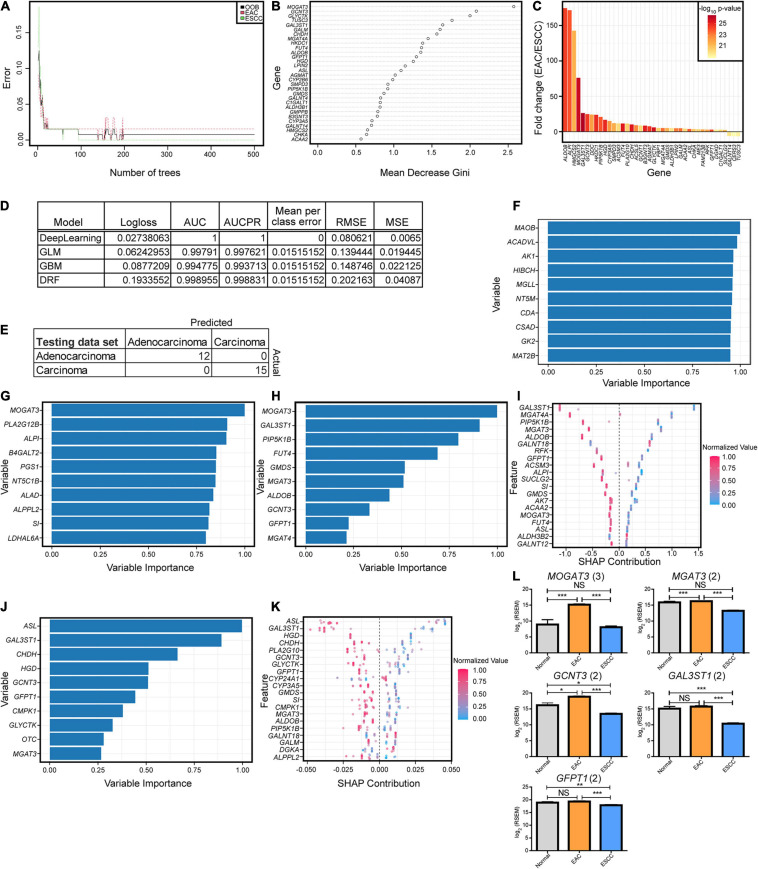
Key enzymatic markers between histological subtypes. **(A)** Random forest error rate with increasing number of trees when differentiating subtypes based on enzymes. **(B)** Random forest variable importance from 500 trees. **(C)** The fold change log_2_ mRNA expression and -log_10_ *p*-values generated from a Mann–Whitney *U* test for the optimal enzymes discovered from the sparse partial least-squares discriminant analysis (sPLS-DA) in Figure 1. **(D)** Machine modules with hyperparameter tuning trained on enzyme expression with the best logloss model being reported for each category, when applicable. Abbreviations are as follows: AUC, area under the curve; AUCPR, area under the precision-recall curve; RMSE, root mean square error; MSE, mean square error. **(E)** All of the models resulted in the same confusion matrix with the same holdout dataset. **(F–K)** Variable importance is seen for each of the top tuned models, including deep learning based on a feedforward neural net **(F)**, generalized linear model **(G)**, gradient boosting machine **(H,I)**, and distributed random forest **(J,K)**. Enzymes reported multiple times in the top 10 for importance in panels **(F–H)** and **(J)** had the expression plotted between the subtypes, with the number in parentheses indicating the number of occurrences **(L)**. Error bars represent the standard error of the mean. Statistics were calculated through Mann–Whitney *U* test. NS, not significant, *p < 0.05, **p < 0.01, ***p < 0.001.

To further examine if the enzyme differences between esophageal cohorts were indeed objectively different, we turned to machine learning approaches. We first examined a simple random forest without hyperparameter tuning aside from increasing the number of trees, which quickly converged before the 500-tree epoch (Figure 2A). The contribution of the genes in the categorical prediction (Figure 2B) was somewhat similar to the 40 genes in component 1 for the sPLS-DA, which can be reasoned by their highly significant differences (Figure 2C). We therefore pursued a variety of hyperparameter-tuned, when appropriate, machine learning approaches that generated great predictive capability in the validation cohort, and all generated the same testing results (Figures 2D,E). Variable contribution for the decision was explored for deep learning, generalized linear model, gradient boosting machine, and distributed random forest (Figures 2F–K). A subset of five genes was repeated across the top 10 most important variables from the models and was found to be significantly different (Figure 2L).

To further examine the malignant transformation into cancerous tissue in hopes of discovering biomarkers, the study examined what genes were most diverged from normal tissue in the transformation to malignant carcinogenesis. To examine their potential use as future biomarkers, genes were included if they showed a 25-fold change when comparing normal to EAC and ESCC, a Benjamini–Hochberg-corrected Mann–Whitney *U* test of *q* < 0.001 between normal tissue and both EAC and ESCC subtypes, and a minimum AUC of 0.95 compared to normal tissue for both groups. Twelve genes appeared, which included two for matrix metallopeptidases (*MMP11*, *MMP12*), homeobox genes (*HOX8*, *HOX10*, *HOX11*), *IL8*, *CHRNA1*, *IBSP*, *HIST1H3B*, *SP8*, *CGB5*, *APOC2*, *C15orf53*, and *CKMT2* (Supplementary Figure 4).

**FIGURE 3:**
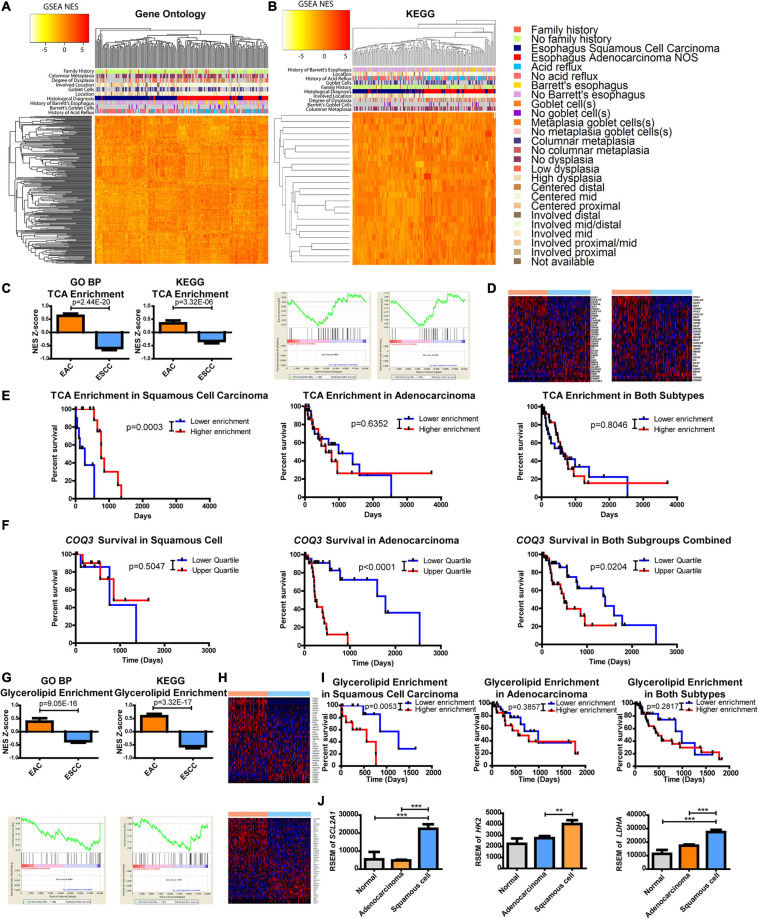
Metabolic pathways are altered between esophageal squamous cell carcinoma (ESCC) and esophageal adenocarcinoma (EAC). Gene set enrichment analysis (GSEA) compared each patient against the average of normal tissues. Normalized enrichment scores (NESs) were plotted and hierarchically clustered for metabolic pathways, defined as ≥10 minimum enzymes with a minimum of 90% of enzymes, for **(A)** all Gene Ontology (GO) classifications and **(B)** metabolic pathways within the Kyoto Encyclopedia of Genes and Genomes (KEGG). **(C)** Each patient’s NES (top) and group average (bottom) for the tricarboxylic acid (TCA) cycle from GO biological process (BP) (left) and KEGG (right) **(D)** along with the heatmaps of the comprising genes within the pathway of GO (top) and KEGG (bottom). **(E)** Patient survival was plotted by separating the upper and lower quartiles of TCA NES through KEGG’s pathway for ESCC (left), EAC (middle), and both (right). **(F)** Patient survival was plotted by separating the upper and lower quartiles of *COQ3* expression in ESCC (left), EAC (middle), and both (right). **(G)** Each patient’s NES (top) and group average (bottom) for glycerolipid enrichment from GO BP (left) and KEGG (right) **(H)** with the heatmaps of the comprising genes within the pathway of GO (top) and KEGG (bottom). **(I)** Patient survival was plotted by separating the upper and lower quartiles of glycerolipid NES through KEGG’s signature for ESCC (left), EAC (middle), and both (right). **(J)** Gene expression within each subtype. Pathway enrichment significance was calculated by a Student’s *t*-test, Mantel–Cox log-rank for survival, and gene expression was compared with a one-way ANOVA with a Bonferroni’s multiple comparison test. **p < 0.01, ***p < 0.001.

Considering the profound metabolic differences observed (Figures 1, 2), esophageal cancer was further examined to identify if the subtypes differed in metabolic pathways, as defined by a gene set comprising a minimum of 10 enzymes, with at least 90% of the genes in the gene set being enzymes. All GO subgroups, including cellular component, BP, and molecular function, were examined through GSEA and revealed a separation of the histological subtypes (Figure 3A). Four metabolic pathway subtypes were identified in EAC and two in ESCC, in which the subtype of EAC impacted survival (Supplementary Figure 5).

The approach was then refined to focus on metabolic pathways by using a smaller database known as KEGG. Results again revealed a separation between EAC and ESCC with enzyme pathways (Figure 3B). There appeared to be a correlation of clinical features associated with histological subtypes in the heatmaps (Figures 3A,B). These correlations were further examined in which histological subtypes had a significant difference in clinical features (Supplementary Figure 6). However, these clinical features failed to show a relationship with subtype-specific enzyme expression with the exception of histological subtype and patients with a history of Barrett’s goblet cell in EAC (Supplementary Figure 7). A relationship with clinical features and survival was also not observed (Supplementary Figure 8). Further validating differences between the subtypes in another dataset provided by the Gene Expression Omnibus, ESCC was found to be more dissimilar to esophageal, gastric, and gastroesophageal adenocarcinomas when comparing only the enzymes or all the genes.

This study sought to further examine differences in metabolic pathways shared among KEGG and GO BP that significantly impact survival. We observed that the tricarboxylic acid (TCA) cycle pathway was noted to be significantly enriched in adenocarcinomas (Figure 3C). The regulation of genes within the TCA pathway was also found to be differentially regulated (Figure 3D). Kaplan–Meier survival curves revealed that the TCA cycle plays a significant role in patient survival for only ESCC compared to EAC, suggesting that the decreased pathway enrichment in ESCC promotes its aggressiveness (Figures 3C,E). These data prompted further examination into TCA-linked energy metabolism. Interestingly, in EAC, there is a very strong survival difference associated with the gene expression of *COQ3*, which involves the electron transport chain (ETC) electron carrier known as ubiquinone or coenzyme Q, but not in ESCC (Figure 3F). Combining these subtypes together dampens the association, as it reveals a weak significant difference. However, considering the significant survival differences, this study next examined which metabolic enzyme had the most significant survival alteration.

The protein-coding gene *COQ3* appeared to be the most significant survival-altering enzyme for EAC, by a large magnitude, compared to the next largest alteration (Supplementary Table 1). On the other hand, ESCC had several enzymes that showed significant correlations with survival, with the largest being *QARS* (Supplementary Table 1 and Supplementary Figure 10). All available genes listed in TCGA and their impact on survival were further examined. Surprisingly, *COQ3* appeared to have the largest significant survival impact on EAC when comparing all available genes, whereas *QARS* ranked number 10 in terms of the *p*-value for EAC (Supplementary Table 2 and Supplementary Figure 10). These data suggest that ESCC is more aggressive when TCA enrichment is decreased. In contrast, EAC appears to function in reverse, being highly dependent on the ETC as seen with *COQ3*, which may allow for specific targeting of subtypes to enhance patient response through survival.

It was further observed that there was a difference in the enrichment and expression of glycerolipid enzymes, as represented by the GO BP pathway glycerolipid catabolic process and glycerolipid metabolism in KEGG (Figures 3G,H). Oddly, it appears that increased enrichment of glycerolipid enzymes significantly enhances the aggressiveness of ESCC but not EAC, which masked survival differences when combining the cohorts (Figure 3I). This study hypothesizes that ESCC has limited glycerolipid metabolism. Supporting this finding, positive regulation of lipid transportation was found to be the second highest increased pathway in GO BP by EAC, showing a 98-fold change in enrichment compared to ESCC, suggesting a potentially limited pool of lipids in ESCC (Supplementary Table 3).

This study next aimed to identify which metabolic pathway is supplying carbon and energy for ESCC. It was observed here that the TCA cycle is enriched in EAC and that increased EAC aggressiveness is associated with the ETC as seen through *COQ3.* Furthermore, decreased enrichment in the TCA cycle increased ESCC aggressiveness (Figures 3C,E,F), suggesting that the ESCC energy flux lies elsewhere. Anaerobic glycolysis is known to be upregulated in many cancer types (Vander Heiden et al., 2009). In turn, this study next examined glucose uptake and metabolism. Differences were observed between EAC and ESCC for *SLC2A1* (the gene for GLUT1 for glucose entry), *HK2* (a limiting step in glycolysis), and *LDHA* (an important exit for glycolysis for NAD+ regeneration) (Figure 3J). This suggests that ESCC receives its energy and carbon from glycolysis, whereas EAC has upregulated oxidative metabolic pathways.

**FIGURE 4:**
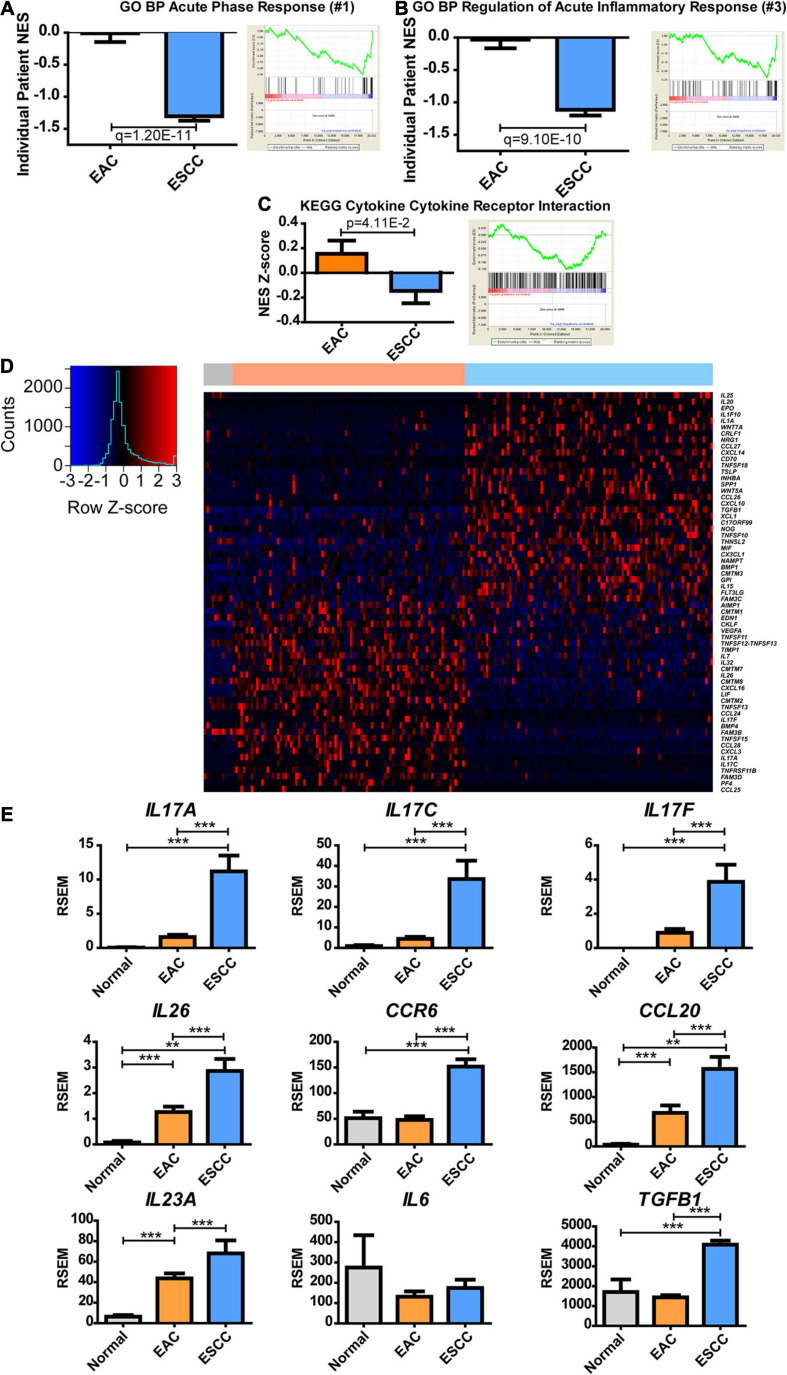
Esophageal squamous cell carcinoma (ESCC) and esophageal adenocarcinoma (EAC) histological subtypes show distinct inflammatory responses. Normalized enrichment score (NES) was calculated for each patient’s tumor by comparing each patient’s expression to the normal adjacent tissue average expression (left) and as a group against the average of each subtype (right) for Gene Ontology biological processes (GO BPs). **(A)** The largest fold change of ESCC compared to EA was discovered to be acute phase response, **(B)** while the third was regulation of acute inflammatory response. **(C)** The same calculation was made for the Kyoto Encyclopedia of Genes and Genomes (KEGG) database to show the NES Z-score (left) and average group enrichment (right). **(D)** Heatmap mRNA Z-scores of differentially expressed genes (Student’s *t*-test, *p* < 0.05) for GO term “cytokine activity” (GO: 0005125). The color bar on top indicates normal (gray), EAC (orange), and ESCC (blue). **(E)** mRNA expression levels for cytokines of interest relating to a Th17 signature. Differences in enrichment scores were calculated by the Student’s *t*-test and corrected with a Benjamini–Hochberg correction when examining multiple pathways in GO. Gene expression was compared with Kruskal–Wallis *H* test with Dunn’s multiple comparison test between all columns. **p < 0.01, ***p < 0.001.

Next, we examined the metabolic subtypes correlated with other biological responses. While positive regulation of lipid transportation was the second greatest pathway increased in EAC for GO BP, the greatest enriched pathway in ESCC showed a 145-fold change during the acute phase response pathway, which contains the genes involved in an acute inflammatory response (Supplementary Table 3). Significance was seen at both the individual and group average level of enrichment (Figure 4A). Further examination of the top hits revealed regulation of acute inflammatory response as the third largest increase in ESCC over EAC with a 36-fold decrease, which was significant for both the individual level and group level of enrichment (Figure 4B and Supplementary Table 3). KEGG does not appear to have a pathway dedicated solely to inflammation but contains a related cytokine–cytokine receptor interaction pathway, which showed marginally significant enrichment (Figure 4C and Supplementary Table 4). Therefore, a heatmap was constructed to examine the role of inflammation and cytokines utilizing the genes contained in GO’s term “cytokine activity” (Figure 4D). Two largely different environments were quickly seen, in which case, it was observed that interleukin (IL)17-related cytokines clustered near each other and were highly upregulated in EAC (Figure 4D). Significant upregulation of *IL17A*, *IL17C*, and *IL17F* was confirmed (Figure 4E). Observing a cytokine-based signature of Th17 cells, we further examined the signature for Th17 effector memory cells (Korn et al., 2009), in which markers were found to be significantly upregulated, including *IL26*, *CCR6*, and *CCL20* (Figure 4E). Consequently, the study next examined if the cytokine environment induced Th17 cells.

IL-23 is known for further inducing Th17 responses (Korn et al., 2009), but *IL23A* was insignificantly upregulated (*p* = 0.06) (Figure 4E). IL-6, along with transforming growth factor (TGF)-β, can induce naive CD4 T-cell maturation into Th17 (Korn et al., 2009). Although *IL6* expression was unchanged, *TGFB1* expression increased in ESCC. It is possible that Th17 is promoted in the presence of increased *TGFB1* with non-diminished *IL6* or that Th17 infiltrates to the tumor, upon which the tumor microenvironment promotes memory differentiation. These data ultimately show a difference in the inflammatory environment of EAC and ESCC, in which the extent can be seen through cytokines, and further indicate a potential difference in the immune environment.

**FIGURE 5:**
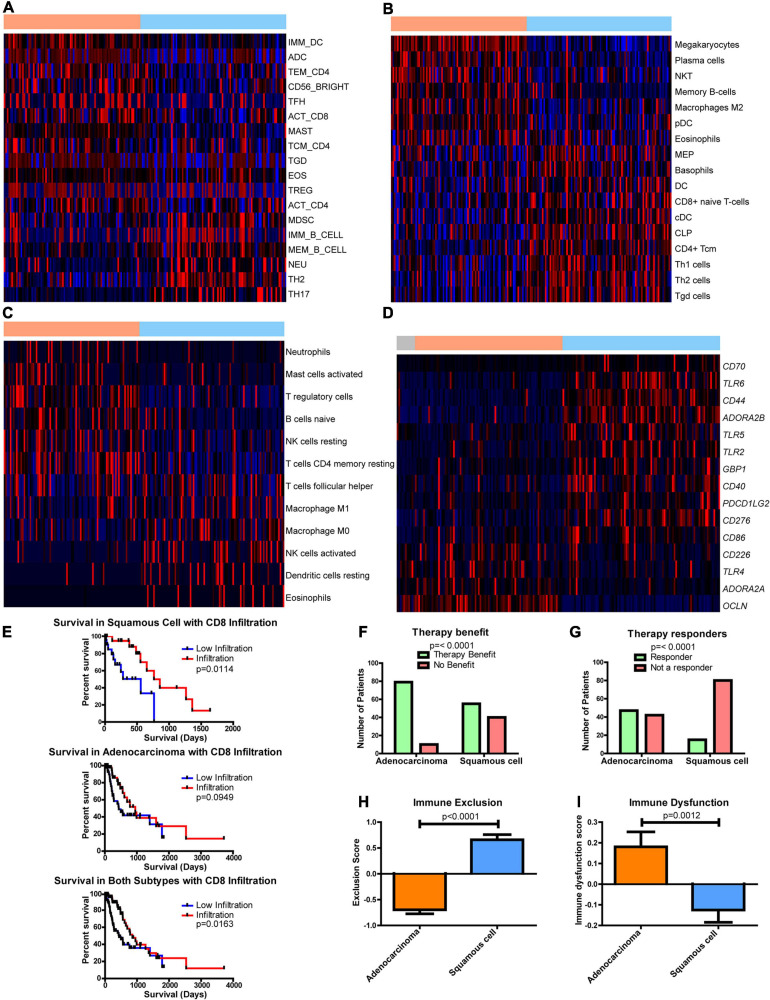
Differences in the immune environment and predicted response to immune therapy between esophageal adenocarcinoma (EAC) and esophageal squamous cell carcinoma (ESCC) histological subtypes. Heatmap Z-scores, ranging from –2 (blue) to 2 (red), of differentially regulated (*q* < 0.05) immune infiltrates according to **(A)** CIBERSORT, **(B)** xCell, **(C)** and TIminer utilizing the dataset of Angelova et al. (2015). **(D)** mRNA Z-scores of differentially expressed (>1.5-fold, *q* < 0.05) genes of potential immunotherapeutic interest between EAC and ESCC. The color bars on top indicate esophageal tissues from normal (gray), adenocarcinomas (orange), and squamous cell carcinoma (blue). **(E)** Kaplan–Meier survival plots for patient survival based on CIBERSORT’s immune prediction. **(F–I)** Shown here are predicted therapy benefit **(F)**, predicted therapy response **(G)**, immune exclusion **(H)**, and immune dysfunction prediction **(I)** according to the computational method of Tumor Immune Dysfunction and Exclusion (TIDE). Survival statistics utilized Mantel–Cox log-rank tests. Therapy prediction utilized Fisher’s exact test and a Student’s *t*-test for immune dysfunction, immune exclusion, and heatmaps, with the latter corrected by Benjamini–Hochberg correction for *q*-values.

To examine if the immune environment is altered between the subtypes EAC and ESCC, this study aimed to bioinformatically validate the cytokine-based hypothesized alteration in Th17 presence and to explore additional alterations in the immune environment. Thus, we utilized TIminer, an algorithm that employs a marker enrichment-based procedure approach to predict immune populations (Tappeiner et al., 2017). The present study then examined Th17 through two independent marker datasets by Angelova et al. (2015) and Charoentong et al. (2017). Both datasets showed a significant increase in Th17 infiltration in EAC compared to ESCC (Supplementary Figure 11), which agreed with the cytokine profile. This study, therefore, expanded to examine the full dataset of Angelova et al. (2015) in TIminer (Figure 5A). Differences were observed between EAC and ESCC, so the number of immune prediction algorithms and prediction-based logic was expanded to further validate and enhance the coverage of immune differences with xCell (Aran et al., 2017), a marker-based approach (Figure 5B), and CIBERSORT (Abbas et al., 2009), which utilizes partial deconvolution to predict the immune populations (Figure 5). An interesting enrichment was observed for dendritic cells (DCs) across all three platforms, as activated DCs, classical DCs, and resting DCs were observed to increase (Supplementary Figures 12A–D). However, CIBERSORT did not show a significant change in activation (Supplementary Figure 12E). Furthermore, the status of CD8 T cells was seen to be significantly increased in ESCC compared to EAC, including signs of naive and activated CD8 T cells (Supplementary Figures 13A,B). However, differences in general CD8 T-cell signatures did not appear significant for xCell (*p* = 0.0543) and CIBERSORT (Supplementary Figures 13C,D). This study further investigated possible markers revealing T-cell status through lymphocyte markers. Markers associated with activation showed a significant increase in ESCC, including *CD44* (Schumann et al., 2015), *CD109* (Haregewoin et al., 1994), and *CD70* (Brugnoni et al., 1997; Tesselaar et al., 2003; Supplementary Figures 13E–G). On the other hand, *PF4* (Supplementary Figure 13H), which is known to inhibit T-cell function (Fleischer et al., 2002), was the second largest significantly downregulated cytokine in ESCC compared to EAC, showing a −54-fold change (Supplementary Table 5). This study further examined helper T cells and immunosuppressive immune cells but did not observe a significant consistent trend (Supplementary Figures 14, 15). Taken together, the strong possibility arises that ESCC represents a potential anti-tumorigenic immune environment through increased DCs and the presence of activated T cells.

The effect of immune presence and response was examined for its potential impact on patient survival. Examining CIBERSORT revealed the presence of CD8 T cells (Figure 5E), and eosinophils had an impact on survival (Supplementary Figure 16). When both subgroups were combined, increased numbers of CD8 T cells were found to enhance survival, but ESCC was seen to have a more significant impact than EAC (Figure 5E). This study utilized the computational framework of TIDE (Jiang et al., 2018) to examine the potential role of checkpoint inhibitors on ESCC and EAC (Jiang et al., 2018). TIDE predicted that EAC will have an enriched benefit and response to checkpoint inhibitor therapy compared to ESCC (Figures 5F,G). Interestingly, TIDE suggests that cytotoxic T lymphocytes are hindered by exclusionary pressures in ESCC and are dysfunctional in EAC (Figures 5H,I). Together with the survival data from CIBERSORT’s predictions, these data suggest that if CD8 T cells can infiltrate into the ESCC tumors, the CD8 T cells can respond positively and promote a survival benefit, whereas CD8 T cells are likely to be dysfunctional in EAC and do not have a significant correlation with survival. Given that EAC likely utilizes dysfunctional mechanisms, it is rationalized that EAC may respond well to checkpoint therapy, which is in agreement with TIDE’s prediction, and could capitalize on the non-exclusionary pressures present in EAC. Furthermore, if the exclusion pressures are reduced in ESCC, ESCC may also show an improved overall survival response, as it is seen to correlate with survival.

As part of this study, literature was searched for genes associated with activation and dysfunction in order to identify therapeutic opportunities. Clear differences were found between EAC and ESCC (Figure 5D). These results include significant differences in the costimulatory B7 molecules of *CD274*, *CD86*, and *CD276* (Supplementary Figures 17A–C), whose protein products bind immune checkpoint molecules PD-1, CLTA4/CD28, and an unknown receptor, respectively (Greaves and Gribben, 2013). Furthermore, differences in *CD40* were observed, as well as in the mRNA encoding its ligand *CD40LG* (Supplementary Figures 17D,E). Interestingly, two of the adenosine receptors, *ADORA2A* (Supplementary Figure 17F) and *ADORA2B* (Supplementary Figure 17G), were significantly increased in EAC and ESCC, respectively. Both receptors have been shown to be stimulated by adenosine and stimulate cAMP production, which plays an immune suppressive role (Sek et al., 2018). The main sources of adenosine in cancer are typically CD39 and CD73, which are encoded by *ENTPD1* and *NT5E*, respectively. A modest decline in *ENTPD1* in ESCC was observed, although EAC had similar levels to the normal adjacent tissue (Supplementary Figure 17H). However, there appears to be an increase in *NT5E* when comparing EAC to ESCC (Supplementary Figure 17I). Together, these data suggest that there are further potential immune-altering targets that are differentially regulated between the histological subtypes, which could potentially be targeted to restore the exclusion and dysfunction seen in ESCC and EAC, respectively.

**FIGURE 6:**
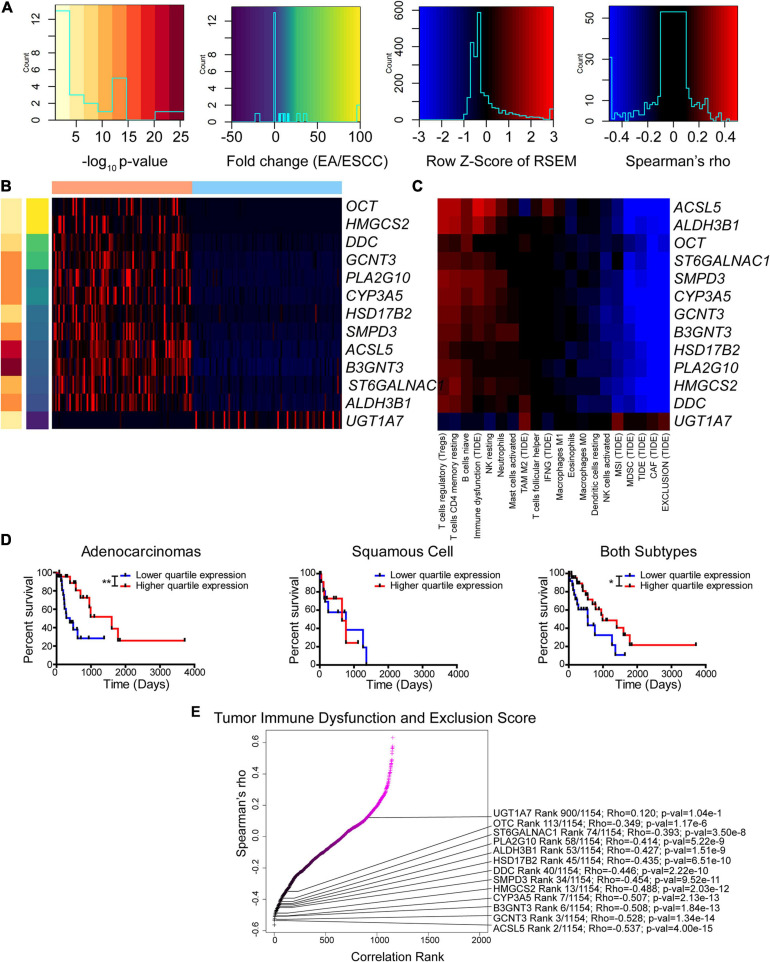
Enzyme expression correlates with immune function prediction. **(A–C)** Legends **(A)** for Z-score of differentially expressed enzymes between subtypes **(B)** and Spearman’s rho for correlation between expression and immune function prediction from CIBERSORT and Tumor Immune Dysfunction and Exclusion (TIDE) utilizing both subtypes **(C)**. **(D)** Kaplan–Meier survival curves for *ALDH3B1*. **(E)** Ranked Spearman’s rho correlation between enzyme mRNA and TIDE score for tumor immune dysfunction and exclusion utilizing both subtypes. Survival statistics utilized Mantel–Cox log-rank. *p < 0.05, **p < 0.01.

Tumor metabolism has been shown to play a significant role in immune recruitment and function (Chang et al., 2015; Allard et al., 2017; Gupta et al., 2017; Rivadeneira and Delgoffe, 2018; Sugiura and Rathmell, 2018; Triplett et al., 2018; Chen et al., 2019; Najjar et al., 2019; Ngwa et al., 2019; Thapa and Lee, 2019; Vigano et al., 2019). To augment the significance of this work, this study investigated if any enzymes are differentially regulated between EAC and ESCC that potentially play a role in immune infiltration. To this extent, 13 enzymes were found to be differentially expressed that correlated with the immune prediction by more advanced algorithms of CIBERSORT and TIDE (Figures 6A–C). The magnitude of the correlation of these genes with the immune environment also appeared to be impacted by the histological subtype (Supplementary Figures 18A,B). It is reasonable to postulate that metabolic differences between subtypes exist and that a given enzyme can influence the environment or *vice versa*.

Of the 13 genes identified, the gene *ALDH3B1* had the greatest association with altered patient survival (Figure 6D) compared to the other genes *CYP3A5*, *UGT1A7*, and *PLA2G10* that were found to be significantly associated with survival (Supplementary Figures 18C–E). The tumor immune dysfunction and exclusion score is postulated to be a strong overall assessment of the immune environment. Therefore, this study examined the 13 enzymes and the association with the scores reported by TIDE (Figure 6E). Many of these differentially expressed genes were found to rank high in their correlation with immune function. The enzyme correlation was then expanded to include all previously used algorithms (Supplementary Figure 19). Likewise, the expanded data support a similar observation, in which seven out of the 13 identified genes, including *CYP3A5*, *PLA2G10*, *UGT1A7*, *GCNT3*, *SMPD3*, *DDC*, and *ST6GALNAC1*, reappeared as top enzymes correlating with the immune environment. These data indicate a casual role of differentially expressed enzymes that correlate with the immune status and highlight the potential role of cancer metabolism in modulating the tumor microenvironment.

## Discussion

Precision medicine holds the promise of correctly leveraging the cellular and extracellular environment against the tumor in order to benefit from unique vulnerabilities. A shift in the metabolic profile of the cell reflects a change in the cellular programming, which is altered through the cancerous transformation. Observing metabolic differences is critical, as the change in the metabolic machinery reflects altered nutrient consumption and production, which may further alter the cell and/or the environment. These differences, ideally, can be targeted through precision medicine.

With the goal of discovering clinical differences that could be targeted to benefit patients, as well as for the effort to discover metabolic differences, we examined multiple cancers in TCGA for clinical attributes including sex, cancer stages, ethnicity, and histology. Interestingly, we did not find key differences in attributes among many cancer cohorts (Supplementary Figures 1, 2). However, we discovered an exception in the histological subtypes for esophageal cancer. These findings were radically different (Figures 1A,B). The differences were so pronounced that some of these enzymes could be used as biomarkers, although better non-enzymatic markers exist to differentiate EAC and ESCC (Supplementary Table 6).

Importantly, there appeared to be a difference in oxidative metabolism between ESCC and EAC. Pathway differences were also found to be associated with survival (Figure 3). EAC is associated with the TCA cycle, glycerolipid metabolism, and shows extreme aggressiveness in patients with increased tumoral expression of electron transport chain gene, *COQ3*. In contrast, ESCC appears to increase glycolysis for energy (Figure 3). These findings mirror a recent study that found lung squamous cell carcinomas drastically increase GLUT1, upregulate glycolysis, and are sensitive to glycolysis modulation compared to lung adenocarcinomas (Goodwin et al., 2017). This difference in GLUT1 expression has also been observed to be largely due to histology, as demonstrated in a meta-analysis between lung squamous cell carcinomas and lung adenocarcinomas (Tan et al., 2017). Recently, it was observed that YAP1 mediates an increase of GLUT1 in esophageal cancer to promote resistance to therapy (Li et al., 2019). This finding raises the question as to how these pathways and survival differences can aid the possible therapeutic intervention to help the prognosis of the patient. Recently, mubritinib cleared a Phase I clinical trial for Erb-B2 receptor tyrosine kinase 2 (*ERBB2*+) solid tumors, which can also inhibit complex I (Baccelli et al., 2019). Although *ERBB2* is seen to significantly impact EAC and both subtypes when combined (Supplementary Table 2), its potential effects could serve a further purpose in EAC by hampering the ETC. However, the treatment of mubritinib for *ERBB2* alone could prove advantageous for EAC, as more genomic alterations are prevalent for *ERBB2* in EAC (23% EAC vs. 3% ESCC) (Wang et al., 2015). Similarly, another study found that 19% of EAC patients have overexpression of *ERBB2* (Dulak et al., 2013). Although trastuzumab, the antibody against *ERBB2*, is already approved for EAC, it is important to consider how far these findings extend to the similarly related gastroesophageal adenocarcinomas (Supplementary Figure 9), gastric adenocarcinomas, and gastroesophageal junction adenocarcinomas, where *ERRB2* may be overexpressed as well (Battaglin et al., 2018). *COQ3* is the most significant survival-altering gene in EAC, which also could be potentially susceptible to the complex I inhibitor metformin (Cameron et al., 2018), which has already been shown to be beneficial in esophageal cancer with combination therapy (Qie et al., 2019). Among this list, *HDAC2* was strongly associated with patient survival in EAC, which also could be potentially targeted with histone deacetylase inhibitors. Furthermore, ESCC could derive its energy from glycolysis, which might be more sensitive to glycolysis inhibitors. These include inhibitors such as 2-deoxyglucose, ionidamine, and silibinin (Sborov et al., 2015), the latter of which was found to decrease *SLC2A1*, *HK2*, and *LDHA* (Shukla et al., 2015), which were observed in the present study to be increased in ESCC (Figure 3J). Our research team previously observed an increased shift from glycolytic to oxidative metabolism under acidic conditions (Abrego et al., 2017). We also observed that acid reflux is enriched in EAC (Supplementary Figure 6A) and hence could drive the potential shift away from glycolysis traditionally observed by the Warburg effect (Vander Heiden et al., 2009) and more toward oxidative metabolism. If the proposed metabolic shift is true, then there is likely to be an impact on response not only to therapies but also to indicators, such as 2-deoxy-2-[^18^F]-fluoro-D-glucose positron emission tomography/computed tomography (Wieder et al., 2004; Jadvar et al., 2006; Higuchi et al., 2008; Roedl et al., 2008; Cuellar et al., 2014; Kim et al., 2019) and NMR (Zhang et al., 2012, 2013). Lastly, these results with the enzymatic differences (Figure 2) and proposed biomarkers (Supplementary Figure 4 and Supplementary Table 6) have the strong potential to be utilized with a device similar to the cytosponge to reduce cost and discomfort associated with traditional endoscopy (Ross-Innes et al., 2015; Heberle et al., 2017; Moinova et al., 2018; Januszewicz et al., 2019).

Differences in glucose metabolism and hypoxic environments have been shown to alter immunity (Barsoum et al., 2014; Noman et al., 2015; Krzywinska and Stockmann, 2018; Li et al., 2018). To this extent, two of the top 3 enriched pathways for ESCC were observed to be associated with inflammation, and there was a drastic change in the cytokine environment (Figure 4 and Supplementary Table 3). Many cytokines are controlled under nuclear factor-κB, which is in part controlled by hypoxia and related stress (Koong et al., 1994; Oliver et al., 2009; Culver et al., 2010; Fitzpatrick et al., 2011; D’Ignazio and Rocha, 2016; Patel et al., 2017). These characteristics are in line with our overall view regarding metabolism and potentially increased hypoxia in ESCC. The data presented here reinforce the hypothesis that metabolic alterations can reveal unique subtypes and their associated selection pressures due to their environments and etiology. The present study further enhances this finding with multiple algorithms to examine the immune status (Figure 5). CD8 T cells and DCs were found to be altered between the subtypes (Supplementary Figures 5, 12, 13), suggesting that adoptive DC therapy or T-cell therapy could have an advantageous role in ESCC treatment. DCs have been associated with an advantage in ESCC patients (Ikeguchi et al., 1998). A previous study examining advanced-stage ESCC observed an immune response with treated monocyte-derived DCs (Narita et al., 2015); however, DCs in ESCC may simply be less suppressed than in EAC (Liu et al., 2009). On the other hand, esophageal cancer, especially ESCC, demonstrates highly expressed (>50%) cancer/testis antigens, including melanoma-associated antigen-A (MAGE-A) (Huang and Fu, 2019). Of note, MAGE-A-specific CD8 T cells can be seen in the peripheral blood of ESCC patients, and these T cells respond to MAGE-A3-loaded DCs to target MAGE-A3+ tumor cells (Huang and Fu, 2019). In the current study, we further observed that CD8 T cells play an important role in survival, similar to a previous observation (Schumacher et al., 2001). Current results also show that EAC is much more likely to respond to checkpoint therapy (Figures 5F,G). However, it is expected that ESCC will show some benefit not only from checkpoint therapy but also, more importantly, from agents geared toward improving immune cell infiltration in tumors, as it has been previously observed that programmed death-ligand 1 (PD-L1) is increased in ESCC and that a decrease in tumor-infiltrating lymphocytes decreases patient survival (Cho et al., 2003; Chen et al., 2016; Yagi et al., 2019).

Recently, the KEYNOTE-028 study utilizing the anti-programmed death-1 (PD-1) antibody pembrolizumab showed that 80% of EAC patients had decreasing tumor volume from baseline compared to 46% in ESCC (Doi et al., 2018). Ultimately, KEYNOTE-181 study showed that targeting PD-L1 was more efficacious than chemotherapy and shows the promise of checkpoint therapy (Shah et al., 2019a). KEYNOTE-180 also showed a great response in ESCC (Shah et al., 2019b), and ultimately, PD-L1 therapy was approved by the Food and Drug Administration to treat ESCC. Although these data highlight the observed correlation of CD8 infiltration and patient survival in ESCC, we suggest that there could be even greater benefit in EAC and caution future studies not to comingle histological subtype covariates. Furthermore, we suggest that there could be further targetable genes and enzymes to help modulate the immune environment (Figures 5, 6 and Supplementary Figures 17–19). Three of these genes belong to the B7 family of ligands, and two of these are adenosine receptors, in addition to adenosine-producing *ENTPD1*. These findings indicate that an improved response is possible for ESCC by utilizing checkpoint inhibition and new combination targets in conjunction with anti-PD-1.

While the immune compartment of the tumor was examined, other components, such as fibroblasts and endothelial cells, could also be pursued for further studies. Additionally, the data analyzed here consisted of the bulk tumor, which merges the heterogeneity of cell types and compartments. As such, important heterogeneity differences, such as regions of hypoxia, which impacts metabolism and the immune response, will be slightly masked. We suggest that the differences are robust and informative and should be further explored with single-cell and spatial transcriptomic approaches to further examine the interplay and heterogeneity between cancer cell subtypes, metabolism, immune cells, and the additional cells present in the stroma.

## Conclusion

Ultimately, we observed a remarkable difference in esophageal metabolism, clinical attributes, cytokines, potential response to therapy, and altered immune and tumor environments between histological subtypes. Unique subtypes were further observed within histological subtypes, which correlated with patient survival and require further examination (Supplementary Figure 5). As such, these data highlight the risks associated with combining histological subtypes for studying esophageal cancer. By separating these vastly different cancers, improved opportunities and options in precision medicine are opened in order to tailor customized therapies suited for these drastic differences.

## Data Availability Statement

The original contributions presented in the study are included in the article/Supplementary Material, further inquiries can be directed to the corresponding author.

## Author Contributions

RK: conception and writing. RK and PS: design and editing performed. RK and FQ: data analysis. FY: statistical supervision. All authors contributed to the article and approved the submitted version.

## Conflict of Interest

The authors declare that the research was conducted in the absence of any commercial or financial relationships that could be construed as a potential conflict of interest.

## Footnotes

1 www.perl.org

2 www.r-project.org

3 https://cibersort.stanford.edu/

4 http://tide.dfci.harvard.edu/

5 https://xcell.ucsf.edu/

6 https://icbi.i-med.ac.at/software/timiner/timiner.shtml

## Abbreviations

AUC: area under the curve
AUCPR: area under the precision-recall curve
BH: Benjamini–Hochberg
BLCA: bladder urothelial carcinoma
BP: biological process
BRCA: breast invasive carcinoma
CAF: cancer-associated fibroblast
COAD: colon adenocarcinoma
DC: dendritic cell
EAC: esophageal adenocarcinoma
ERBB2+: Erb-B2 receptor tyrosine kinase 2
ESCA: esophageal cancer
ESCC: esophageal squamous cell carcinoma
FC: fold change
GO: Gene Ontology
GSEA: Gene Set Enrichment Analysis
KEGG: Kyoto Encyclopedia of Genes and Genomes
KIRC: kidney clear cell renal carcinoma
KIRP: kidney renal papillary carcinoma
LOO: leave-one-out
LUAD: lung adenocarcinoma
LUSC: lung squamous cell carcinoma
MAGE-A: melanoma-associated antigen-A
MSE: mean square error
NES: normalized enrichment score
PAAD: pancreatic adenocarcinoma
PD-1: programmed death-1
PD-L1: programmed death-ligand 1
PLS-DA: partial least-squares discriminant analysis
PRAD: prostate adenocarcinoma acinar type
READ: rectum adenocarcinoma
RMSE: root mean square error
ROC: receiver operating characteristic
RSEM: RNA sequencing by expectation-maximization
sPLS-DA: sparse partial least-squares discriminant analysis
STAD: stomach adenocarcinoma
TCA: tricarboxylic acid
TCGA: The Cancer Genome Atlas
TIDE: Tumor Immune Dysfunction and Exclusion
TIminer: tumor immunology miner
UCEC: uterine corpus endometrial carcinoma.
