# Supplementary material for: Metabolic and Immunological Subtypes of Esophageal Cancer Reveal Potential Therapeutic Opportunities
Source: Front Cell Dev Biol. 2021 Jul 8;9:667852. doi: 10.3389/fcell.2021.667852 (PMC8295652; doi:10.3389/fcell.2021.667852)
Supplement: Supplementary file 1 [file Data_Sheet_1.PDF]

Supplementary Figures and Legends

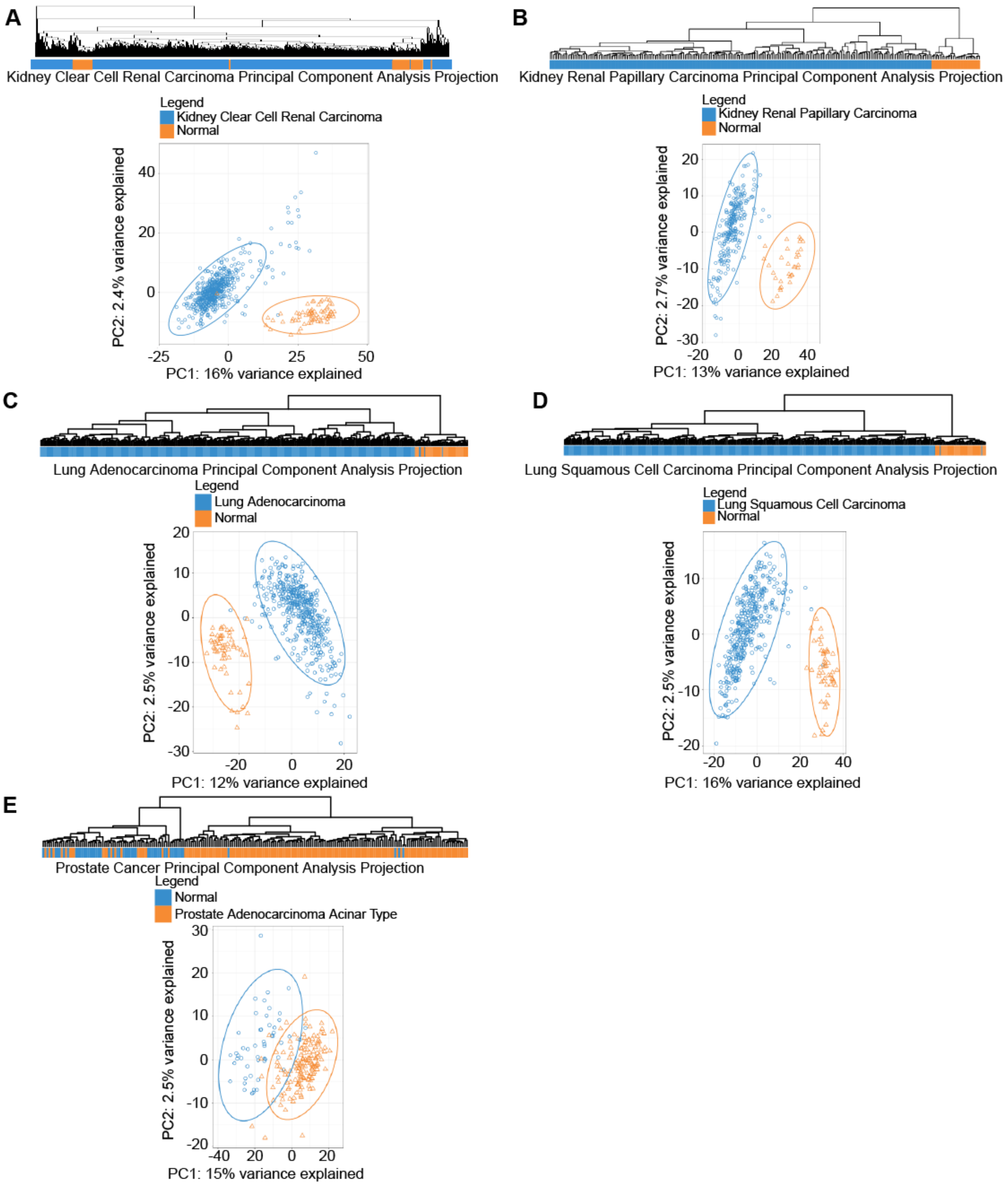

Supplementary Figure 1: Normal enzyme expression differs from cancerous tissue. A total of 13 cancers were screened to determine if clinical features can be used to separate enzyme expression in TCGA cohorts. These five TCGA cancer cohorts contained only one histological subtype in addition to normal tissue after filtering for a minimal sample size of five. Enzyme mRNA

PLS-DA and hierarchical clustering of primary tissue with normal adjacent tissue for (A) kidney clear cell renal carcinoma (KIRC), (B) kidney renal papillary carcinoma (KIRP), (C) lung adenocarcinoma (LAUD), (D) lung squamous cell carcinoma (LUSC), (E) and prostate adenocarcinoma acinar type (PRAD).

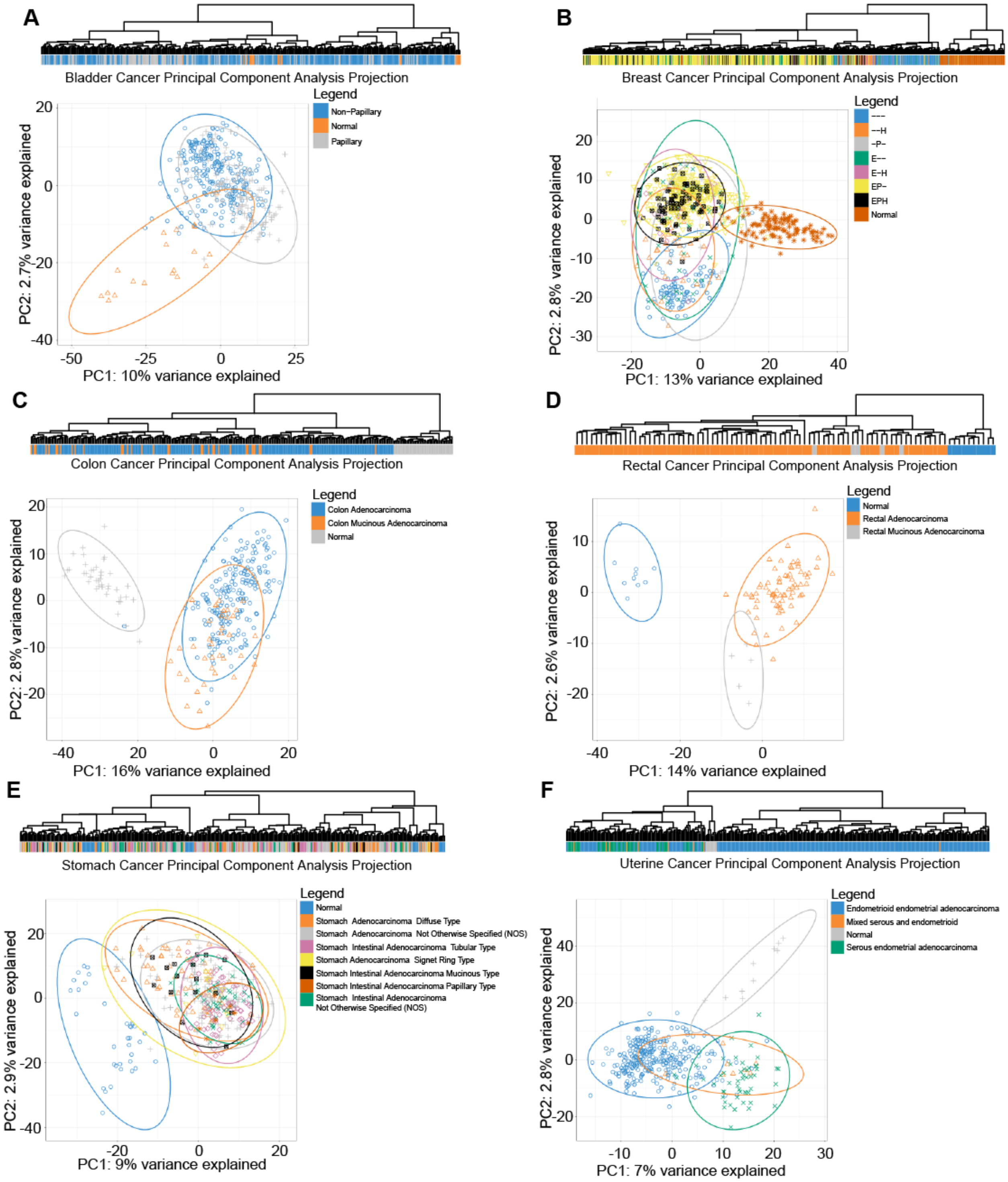

Supplementary Figure 2: Cancers from the screen show that most histological subtypes cannot be separated by enzyme expression. The 13 cancers were screened to determine if clinical features can be used to separate enzyme expression. Shown here are the remaining tissues in the screen, except for esophageal cancer (ESCA) and pancreatic adenocarcinoma (PAAD), the latter was removed due to insufficient normal tissue (n = 4). PLS-DA and hierarchical clustering of enzyme mRNA expression for TCGA cohorts (A) colon adenocarcinoma (COAD), (B) rectum adenocarcinoma (READ), (C) bladder urothelial carcinoma (BLCA), (D) breast invasive

carcinoma (BRCA), (E) stomach adenocarcinoma (STAD), (F) and uterine corpus endometrial carcinoma (UCEC).

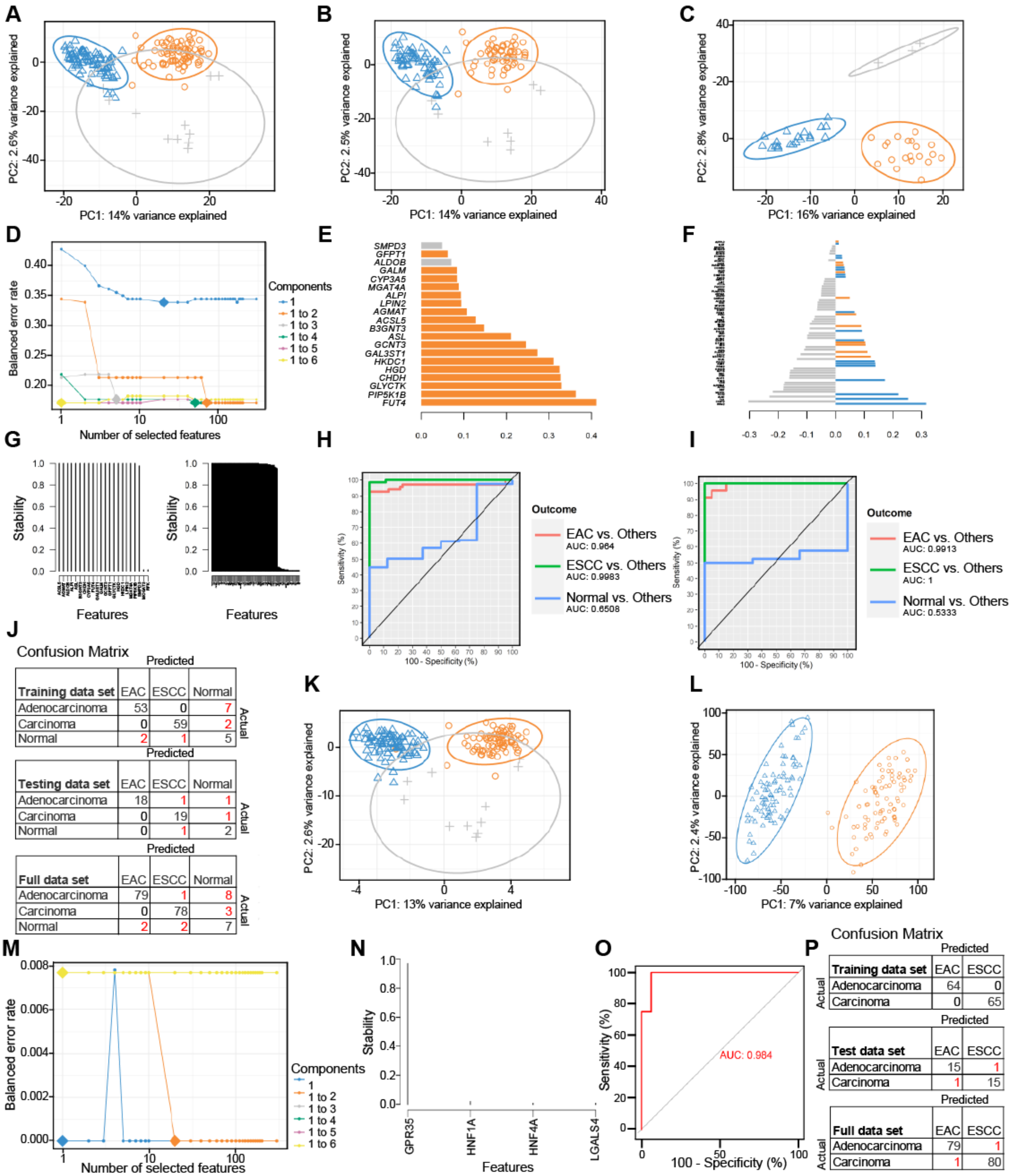

Supplementary Figure S3: Machine learning separates esophageal cancers and adjacent normal tissue by enzyme expression. sPLS-DA tuning was carried out to test the ability of enzyme expression profiles to distinguish normal adjacent tissue from cancerous tissue. (A-C) PLS-DA of the overall (A), training (B), and testing datasets (C). The top six components with either 1-10, 20, 30, 40, 50, 60, 70, 80, 90, 100, 110, 120, 130, 140, 150, 160, 170, 180, 190, 200, 300 enzymes contributing to the component. Eigenvalues ("enzyme weights") contributing to component 1 (E) and component 2 (F), along with their stability (G). (H-I) Receiver operating

characteristic curve utilizing the sPLS-DA for the training (H) and testing dataset (I). The confusion matrix for the outcome of the tuned sPLS-DA classification (J). The resulting sPLS-DA with all of the data (K). All of the mRNA encoding genes were examined on a PLS-DA (L) and optimal combination of components and variables (M). The stability of the first component's single gene plotted alongside additional genes (N). The resulting ROC from the testing dataset (O) and confusion matrix from utilizing sPLS-DA classification with a single gene in a single component (P).

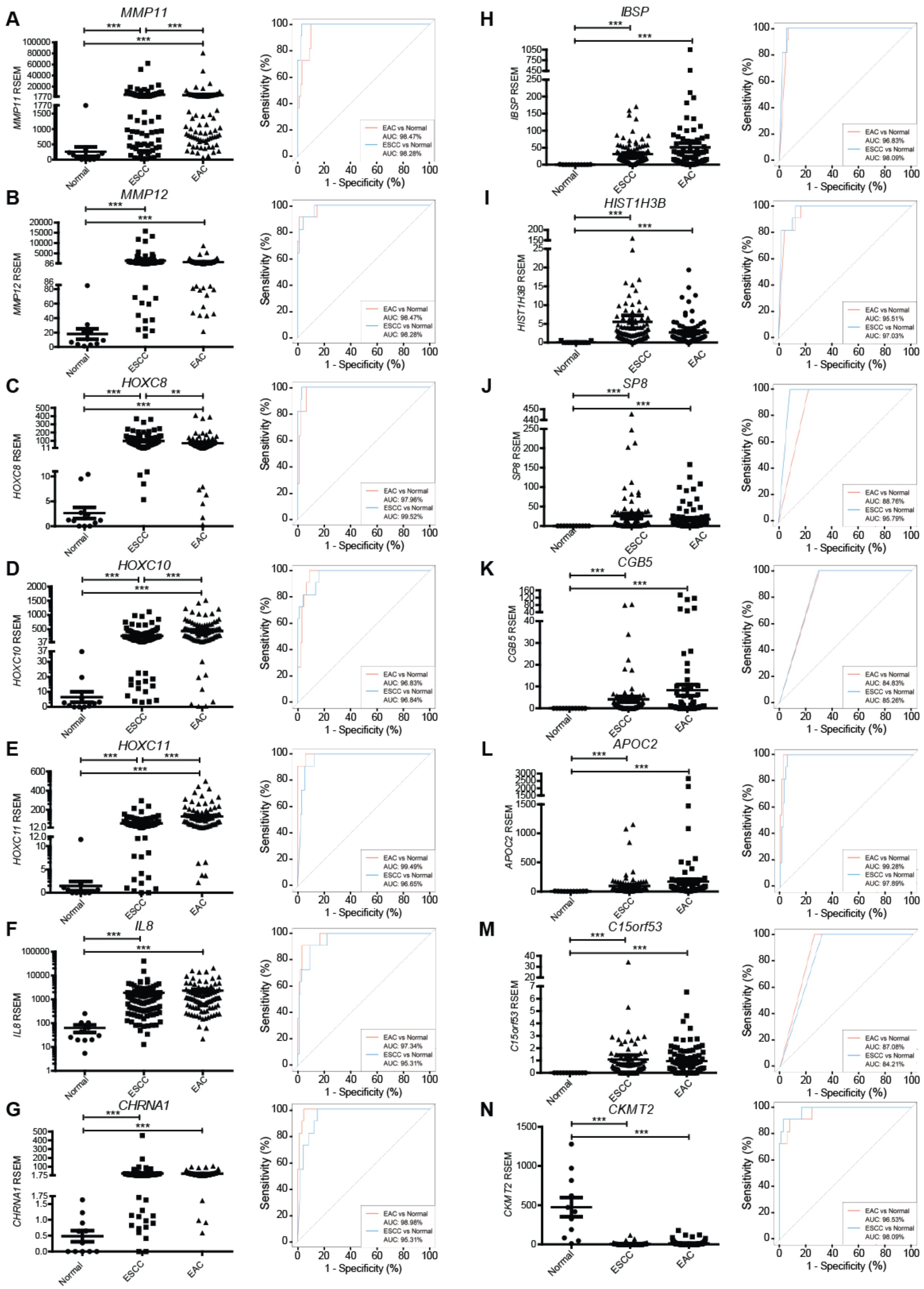

A
MMP11
MMP11 RSEM
Normal
ESCC
EAC
Sensitivity (%)
1 - Specificity (%)
EAC vs Normal
AUC: 98.47%
ESCC vs Normal
AUC: 98.28%
B
MMP12
MMP12 RSEM
EAC vs Normal
AUC: 98.47%
ESCC vs Normal
AUC: 98.28%
C
HOXC8
HOXC8 RSEM
EAC vs Normal
AUC: 97.96%
ESCC vs Normal
AUC: 99.52%
D
HOXC10
HOXC10 RSEM
EAC vs Normal
AUC: 96.83%
ESCC vs Normal
AUC: 96.84%
E
HOXC11
HOXC11 RSEM
EAC vs Normal
AUC: 99.49%
ESCC vs Normal
AUC: 96.65%
F
IL8
IL8 RSEM
EAC vs Normal
AUC: 97.34%
ESCC vs Normal
AUC: 95.31%
G
CHRNA1
CHRNA1 RSEM
EAC vs Normal
AUC: 98.98%
ESCC vs Normal
AUC: 95.31%
H
IBSP
IBSP RSEM
EAC vs Normal
AUC: 96.83%
ESCC vs Normal
AUC: 98.09%
I
HIST1H3B
HIST1H3B RSEM
EAC vs Normal
AUC: 95.51%
ESCC vs Normal
AUC: 97.03%
J
SP8
SP8 RSEM
EAC vs Normal
AUC: 88.76%
ESCC vs Normal
AUC: 95.79%
K
CGB5
CGB5 RSEM
EAC vs Normal
AUC: 84.83%
ESCC vs Normal
AUC: 85.26%
L
APOC2
APOC2 RSEM
EAC vs Normal
AUC: 99.28%
ESCC vs Normal
AUC: 97.89%
M
C15orf53
C15orf53 RSEM
EAC vs Normal
AUC: 87.08%
ESCC vs Normal
AUC: 84.21%
N
CKMT2
CKMT2 RSEM
EAC vs Normal
AUC: 96.53%
ESCC vs Normal
AUC: 98.09%

Supplementary Figure S4. Biomarkers to differentiate cancerous tissue from normal adjacent tissue. ROC curves showing the performance of single enzymes to differentiate normal tissue from cancerous tissue in esophageal cancer. Only the best performing genes separating normal from cancerous tissue were plotted, i.e. *MMP11* (A), *MMP12* (B), *HOXC8* (C), *HOXC10* (D), *HOXC11* (E), *IL8* (F), *CHRNA1* (G), *IBSP* (H), *HIST1H3B* (I), *SP8* (J), *CGB5* (K), *APOC2* (L), *C15orf53* (M), and *CKMT2* (N) are represented that were characterized by a fold change greater than 25 with a Benjamini-Hochberg's corrected q < 0.001 after Mann–Whitney U test for both subtypes compared to normal tissue. Statistics denote Kruskal–Wallis H test with Dunn's multiple comparison test between all columns. **P < 0.01, ***P < 0.001.

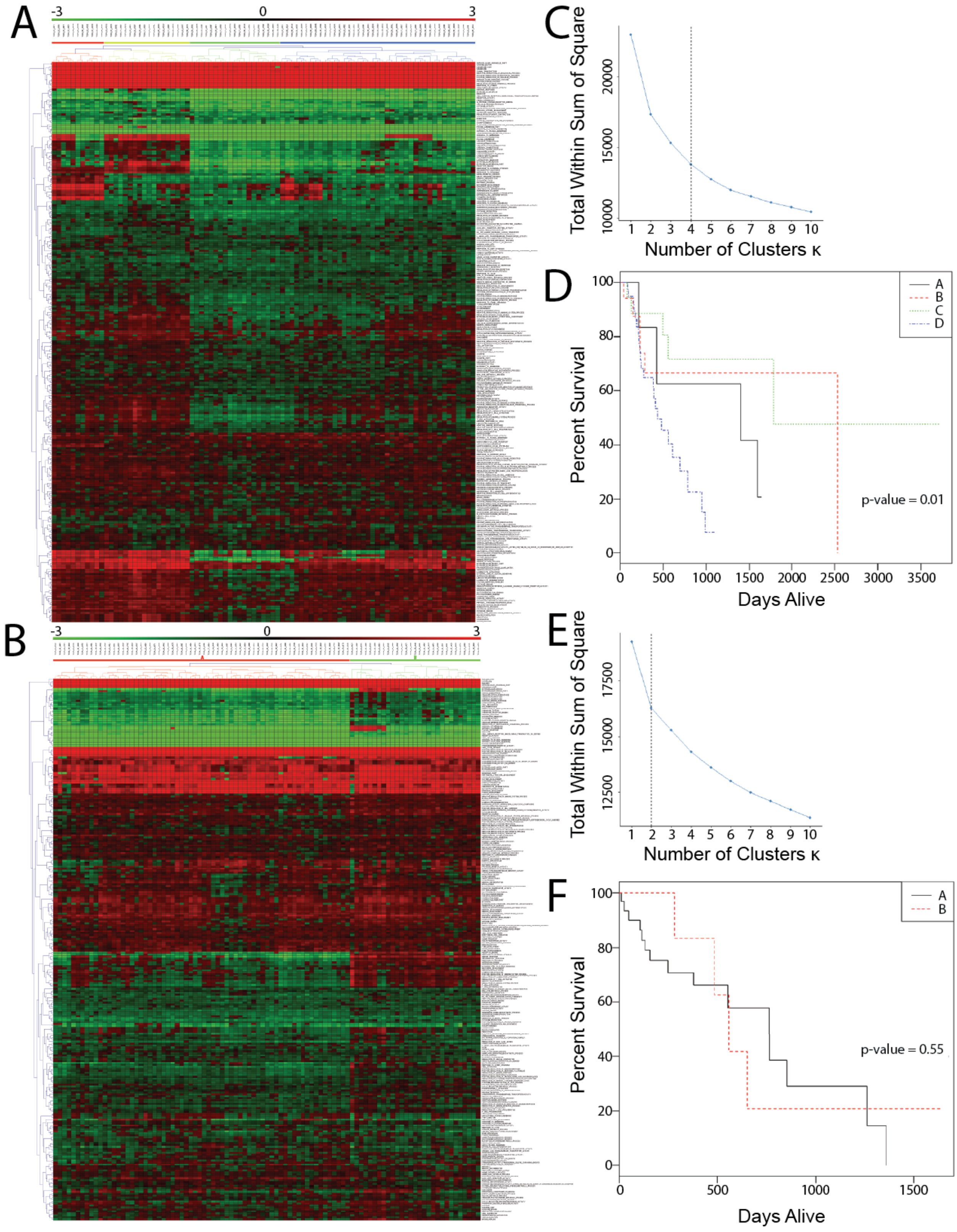

Supplementary Figure 5. Metabolic-associated pathways show subtypes within histological subtypes. Each patient had their gene enrichment score calculated by comparing their expression to the average of normal adjacent tissue for all gene ontology terms. Pathways

were then filtered if they were deemed to be metabolically associated (n ≥ 10 enzymes with ≥ 90% of genes being enzymes) for (A) EAC and (B) ESCC. (C) EAC with hierarchical cluster analysis with Ward's minimum-variance method was used to classify patients into clusters, upon which the number of clusters was preceded by the index of within-cluster sums of squares. (D) Kaplan-Meier curve for EAC clusters was followed by a log-rank test for significance. (E) Cluster analysis was performed for ESCC and (F) the resulting Kaplan-Meier curve of ESCC clusters.

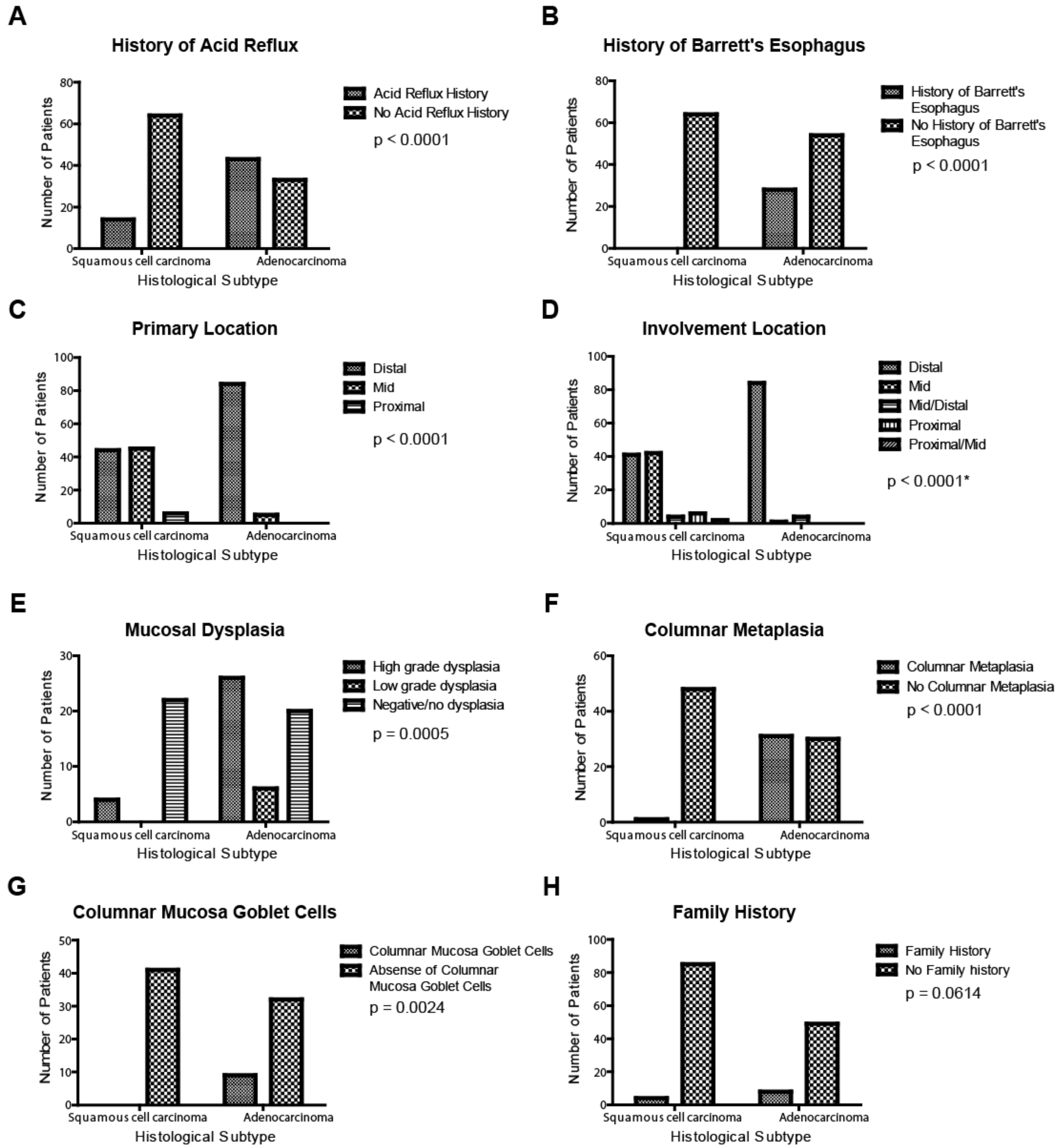

Supplementary Figure 6. Clinical features associated with esophageal histological subtypes. Clinical features were examined for their occurrence within both subtypes for (A) acid reflux, (B) Barret's esophagus, (C) primary location, (D) involved location, (E) mucosal dysplasia, (F) columnar metaplasia, (G) columnar mucosa goblet cells, and (H) family history. Statistics were calculated using Fisher's exact test when only two categories existed, and the Chi-squared method was used when there are more than two categories. The asterisk (*) denotes when Proximal and Proximal/Mid was removed from the statistical calculation as it had no patients in these categories for adenocarcinoma, which invalidates the Chi-squared test.

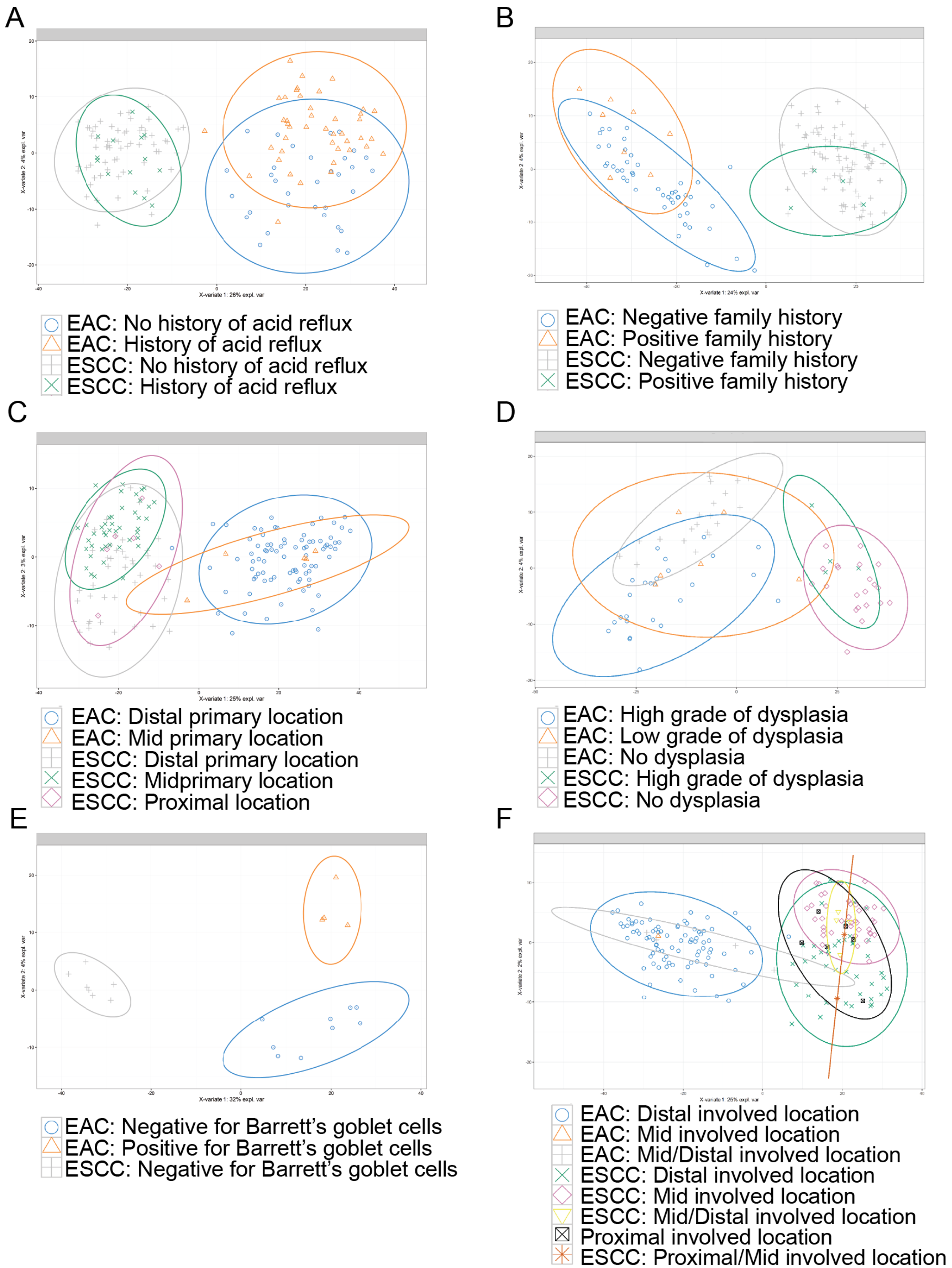

Supplementary Figure 7. Clinical feature association with enzyme expression. Clinical

features were examined with PLS-DAs for their occurrence within both subtypes to determine if these clinical features could influence enzyme mRNA expression in connection with (A) acid reflux, (B) family history, (C) primary location, (D), degree of dysplasia, (E) Barrett’s goblet cells, and (F) involved location.

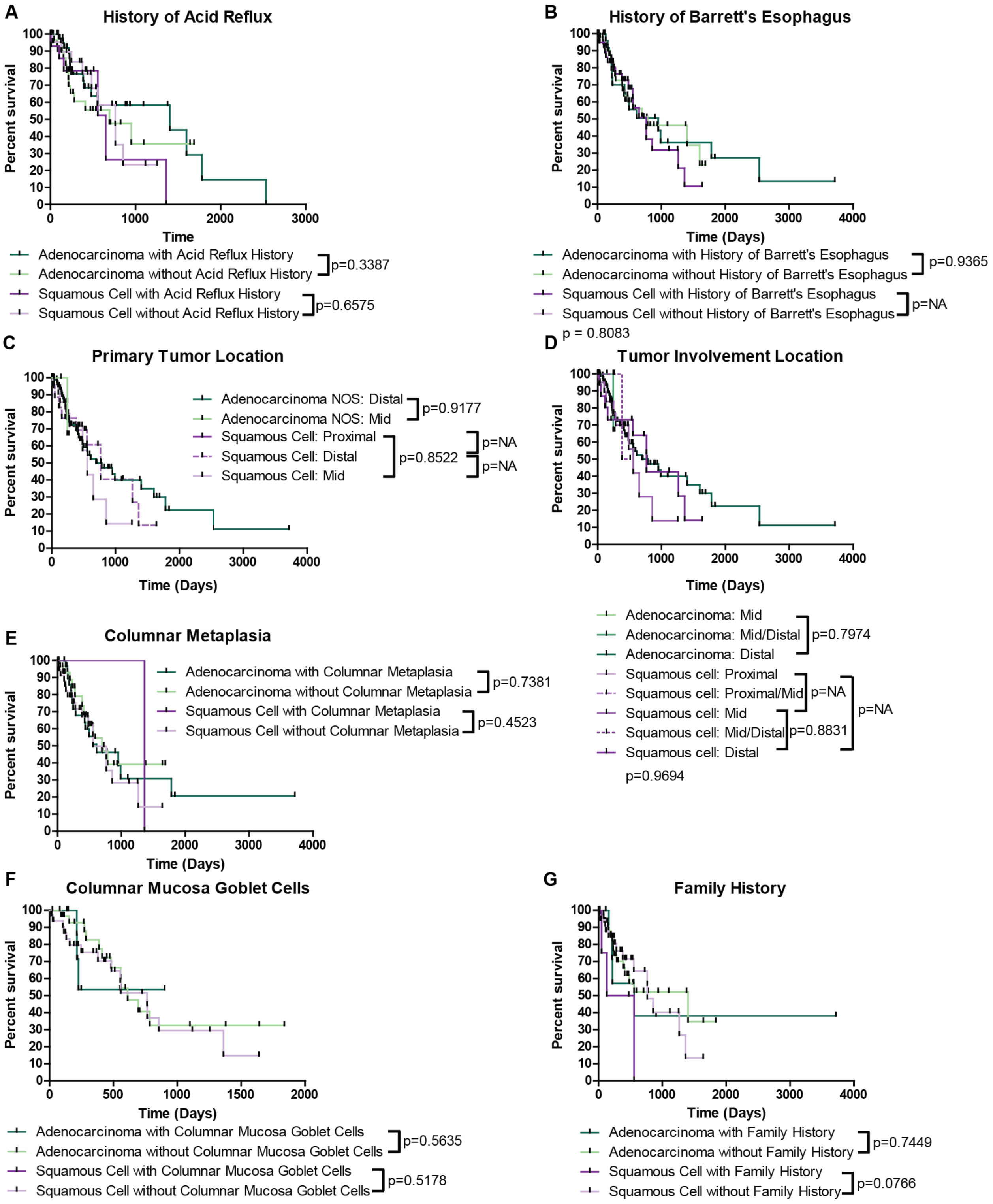

Supplementary Figure 8. Clinical feature association with survival. Clinical features within histological subtypes were examined based on their impact on survival. Kaplan-Meier curves were plotted for (A) history of acid reflux, (B) history of Barrett's esophagus, (C) primary tumor location, (D) tumor involvement location, (E) columnar metaplasia, (F) columnar mucosa goblet cells, and (G) family history. The Mantel-Cox log-rank test was used to calculate significance.

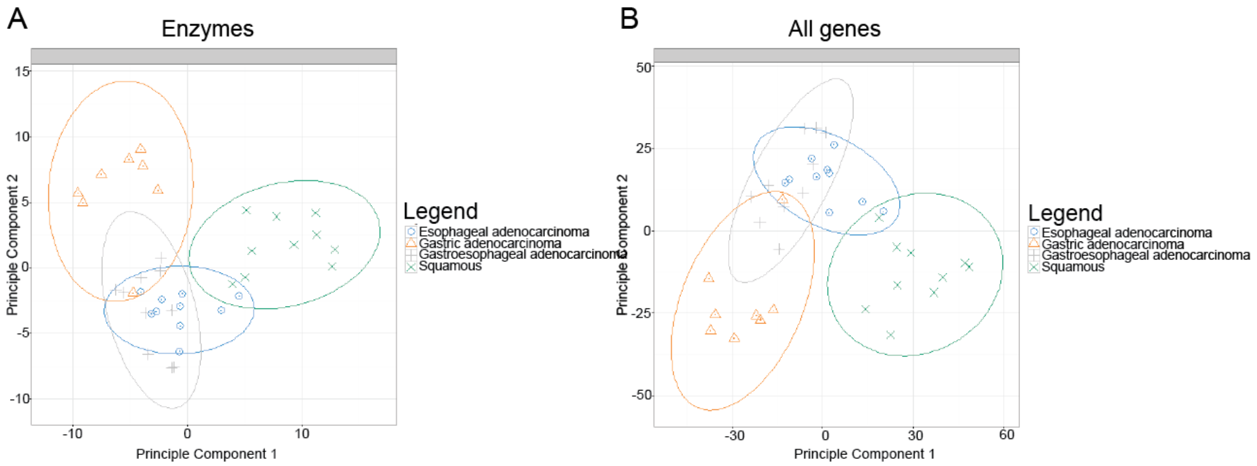

Supplementary Figure 9. Independent study to confirm separation between subtypes. Gene Expression Omnibus accession GSE27040 was examined to determine if separation between subtypes was observable in an independent project. Shown here is the PLS-DA of mRNA expression for (A) enzymes and (B) all available genes examined.

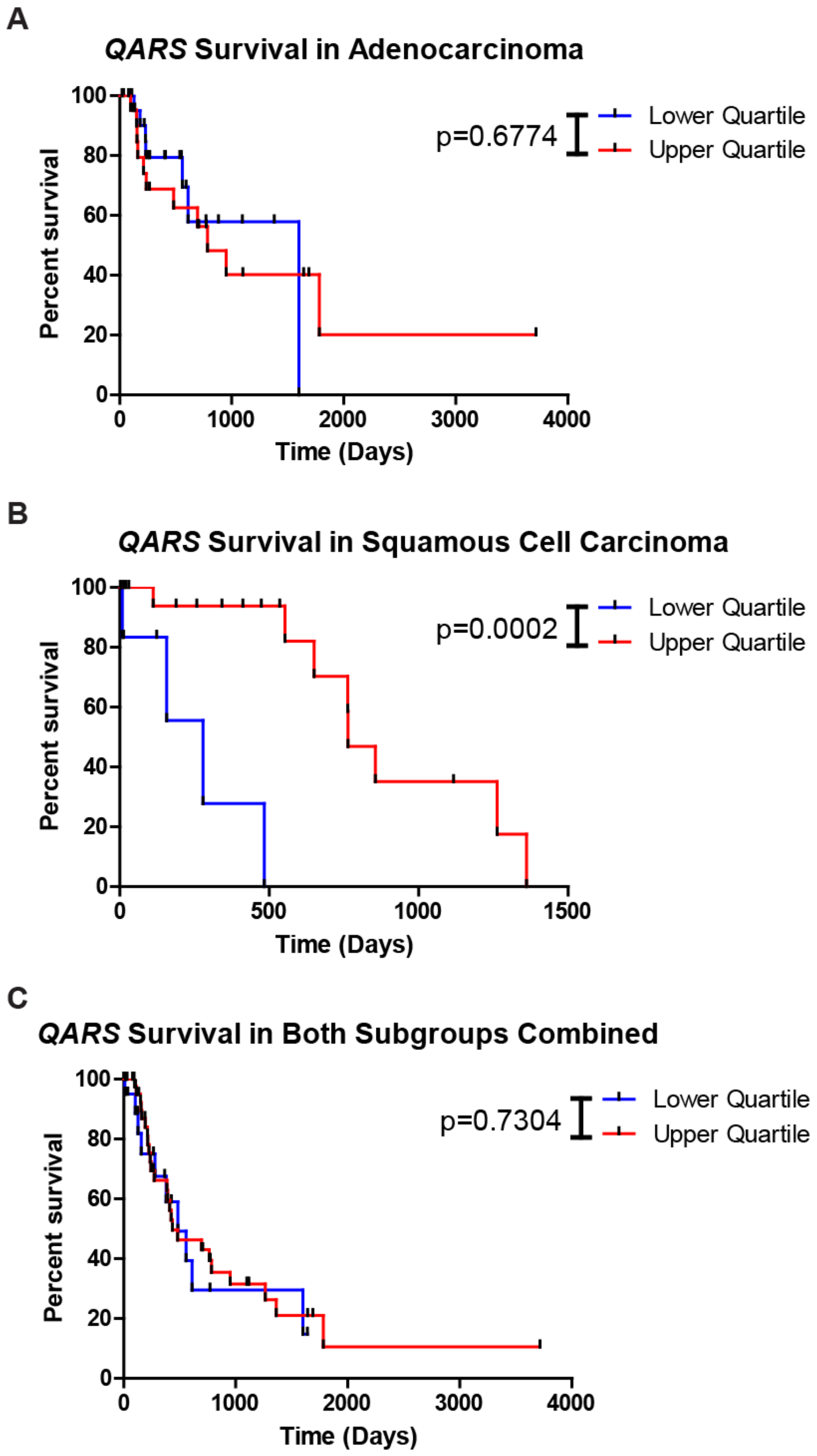

Supplementary Figure 10. *QARS* impact on survival in esophageal cohorts. Shown here are Kaplan-Meier curves to examine the survival of patients, applying upper and lower quartile ranges of *QARS* mRNA expression in (A) EAC, (B) ESCC, and (C) combined subtypes. The Mantel-Cox log-rank test was used to calculate significance.

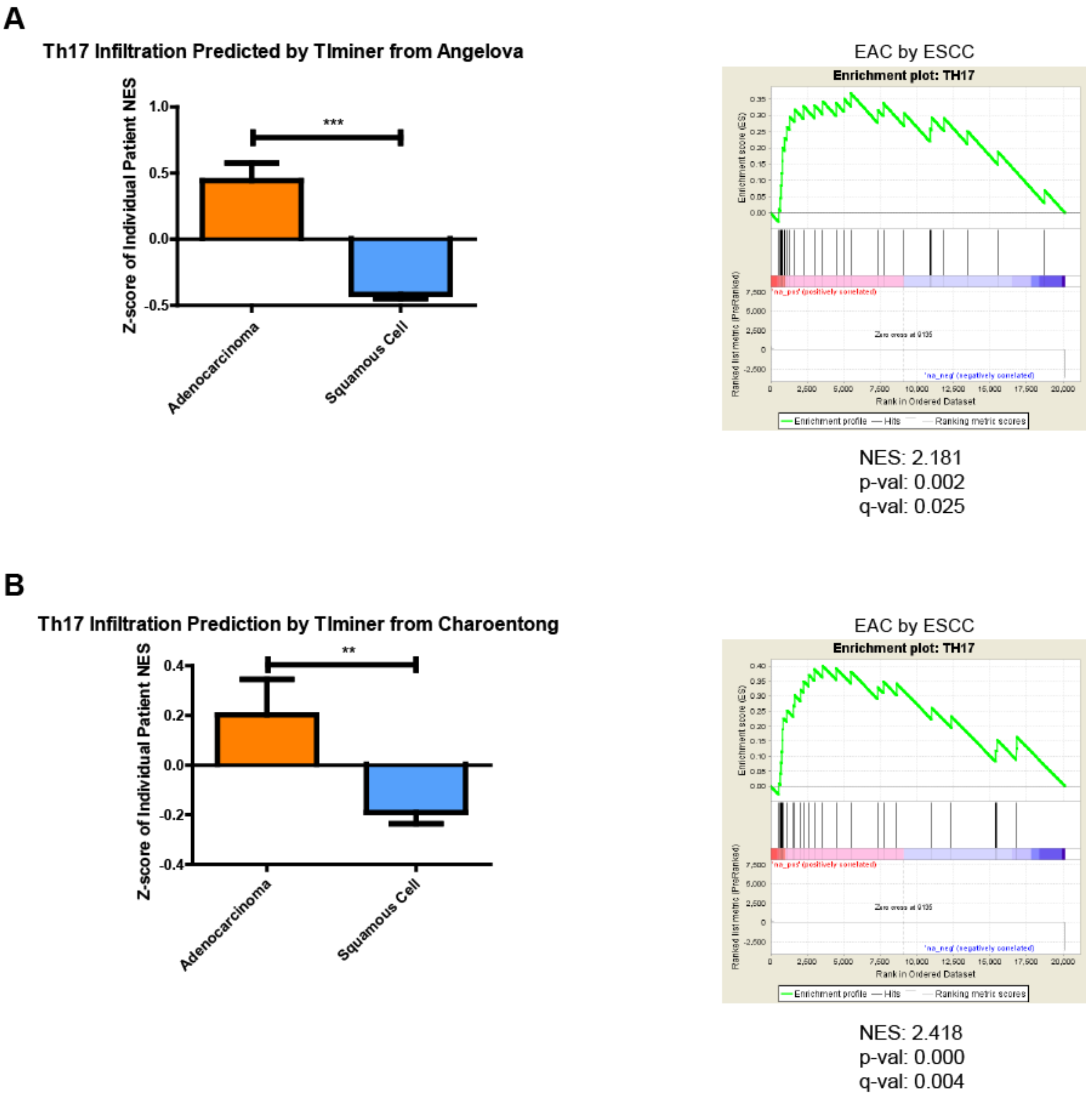

Supplementary Figure S11. Prediction of Th17 infiltration in histological subtypes. (A-B) Validation of the indication of Th17, as portrayed by cytokines, was examined through TIminer [38], with the signature supplied by Angelova (A) [39] and Charoentong (B) [40]. Provided here are Z-scores of each patient's normalized enrichment score generated by gene set enrichment analysis (GSEA) when compared to normal tissue (left) and the chart when comparing to the average of adenocarcinoma group and to the average of the squamous cell carcinoma group (right). The GSEA chart was calculated by dividing the average group RSEM for EAC by ESCC. Statistics supplied by GSEA utilized the q-value from Benjamini-Hochberg's correction. Student's t-test was conducted for significance for bar charts. **P < 0.01, ***P < 0.001.

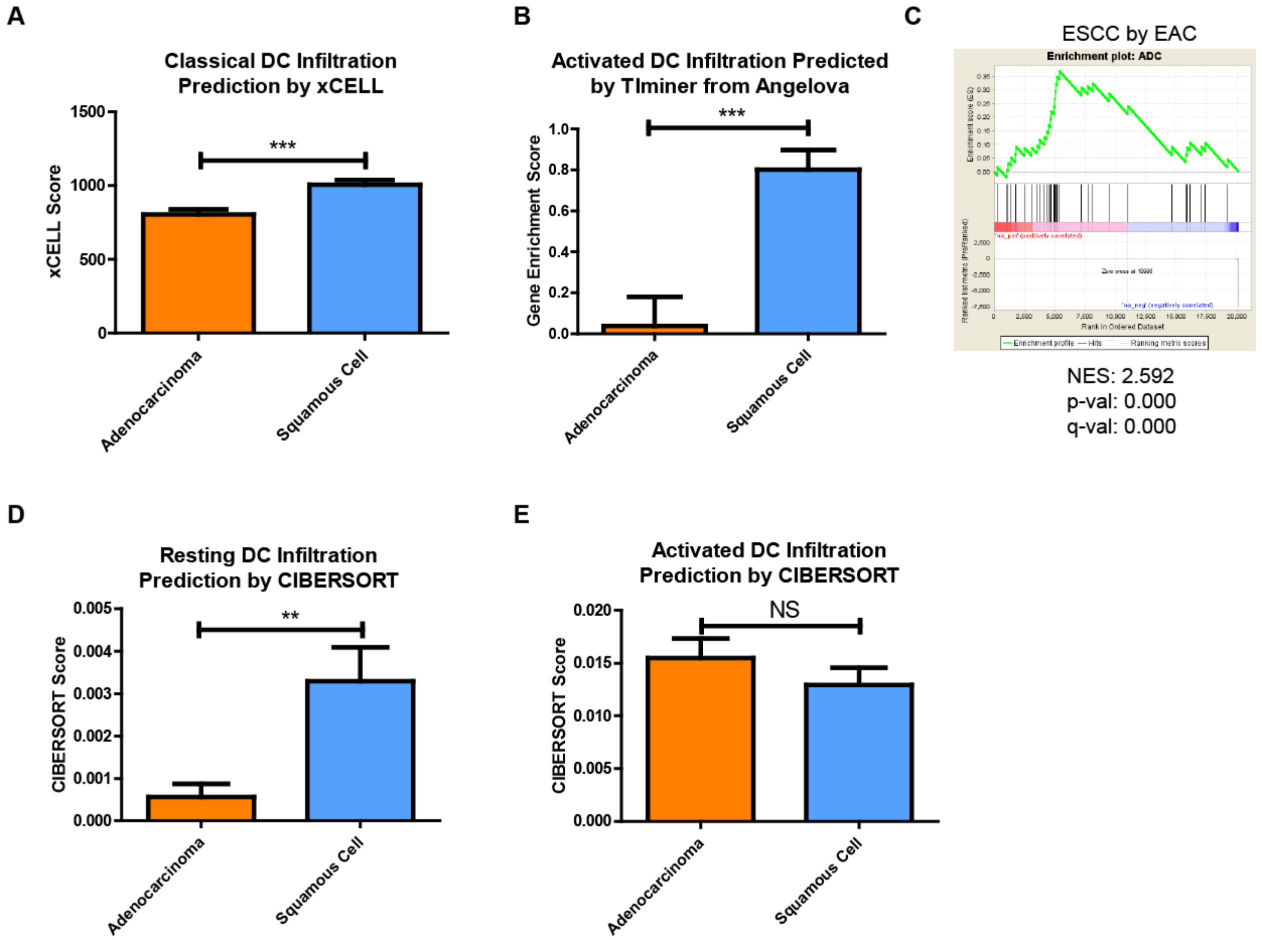

Supplementary Figure S12. Dendritic cell prediction in histological subtypes. Dendritic cell (DC) immune status was predicted through several algorithms. This includes classical DC infiltration from xCell (A), activated DCs from Angelova by TIminer (B) and the resulting group enrichment plot (C), resting DC infiltration (D), and activated DC infiltration by CIBERSORT (E). Gene set enrichment analysis (GSEA) bar chart was calculated by dividing each patient's RSEMs by the average normal tissue to generate the normalized enrichment score (NES). The GSEA graph compares the average RSEM for ESCC to the average RSEM of EAC. Statistics supplied by GSEA with q-value indicating Benjamini-Hochberg's correction. Student's t-test was conducted for significance for bar charts. **P < 0.01, ***P < 0.001. NS: not significant.

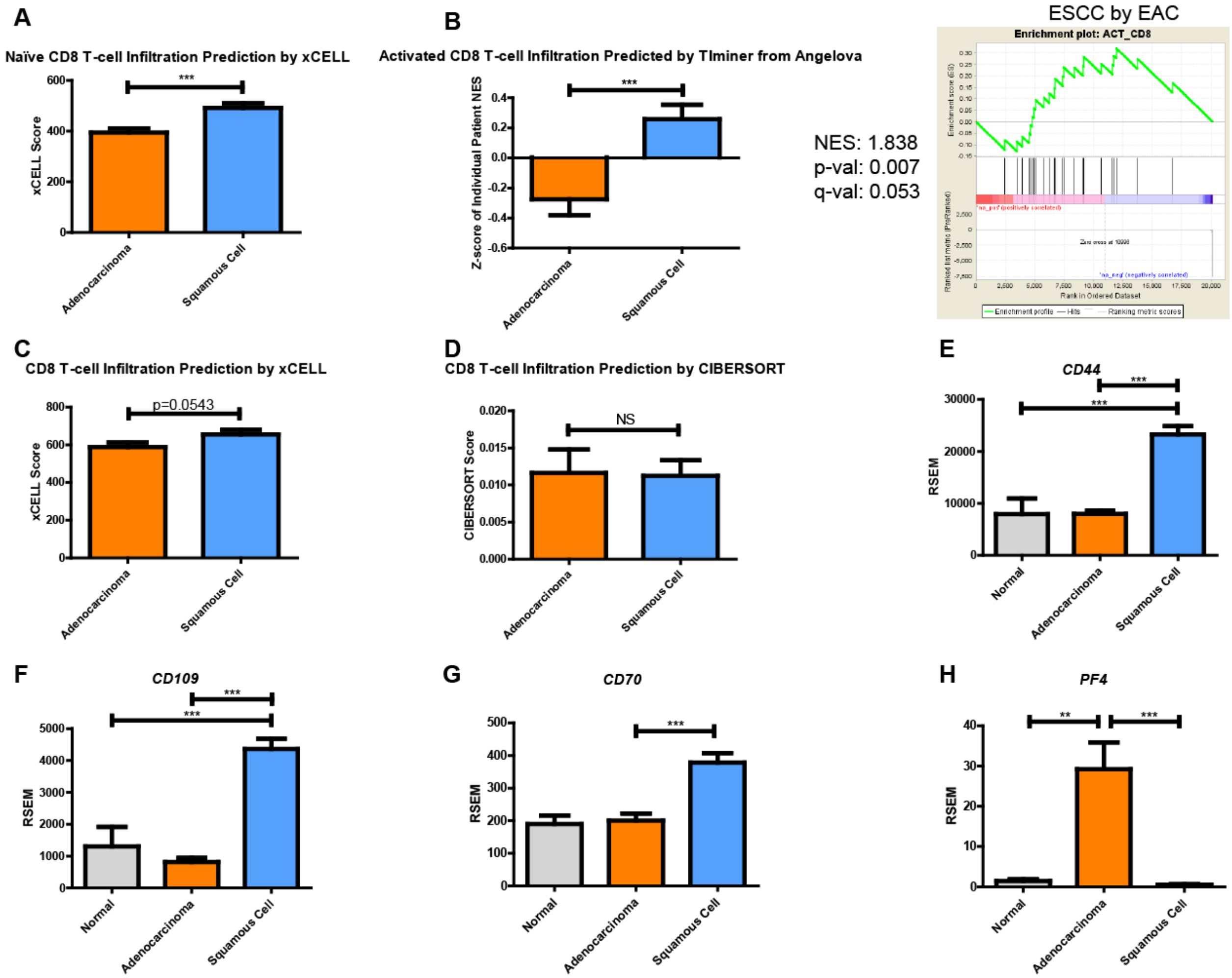

Supplementary Figure 13. CD8 T-cell prediction in histological subtypes. CD8 T-cell immune status was predicted through several algorithms. This includes (A) naïve CD8 T-cell infiltration by xCell, (B) Angelova's signature for activated CD8 T-cell infiltration by TIminer, including individually calculated Z-scores (left) and group comparison between subtypes (right), (C) CD8 T-cell infiltration by xCell, (D) and CD8 T-cell infiltration by CIBERSORT. mRNA expression was further examined for clues about CD8 T-cell status, including (E) *CD44*, (F) *CD109*, (G) *CD70*, (H) and *PF4*. Gene set enrichment analysis (GSEA) was calculated by dividing the group average RSEM for ESCC by EAC. Statistics supplied by GSEA with q-value indicating Benjamini-Hochberg's correction. A Student's t-test was conducted for significance for immune infiltration and a one-way ANOVA with Bonferroni's multiple comparison test for mRNA expression. ***P < 0.001.

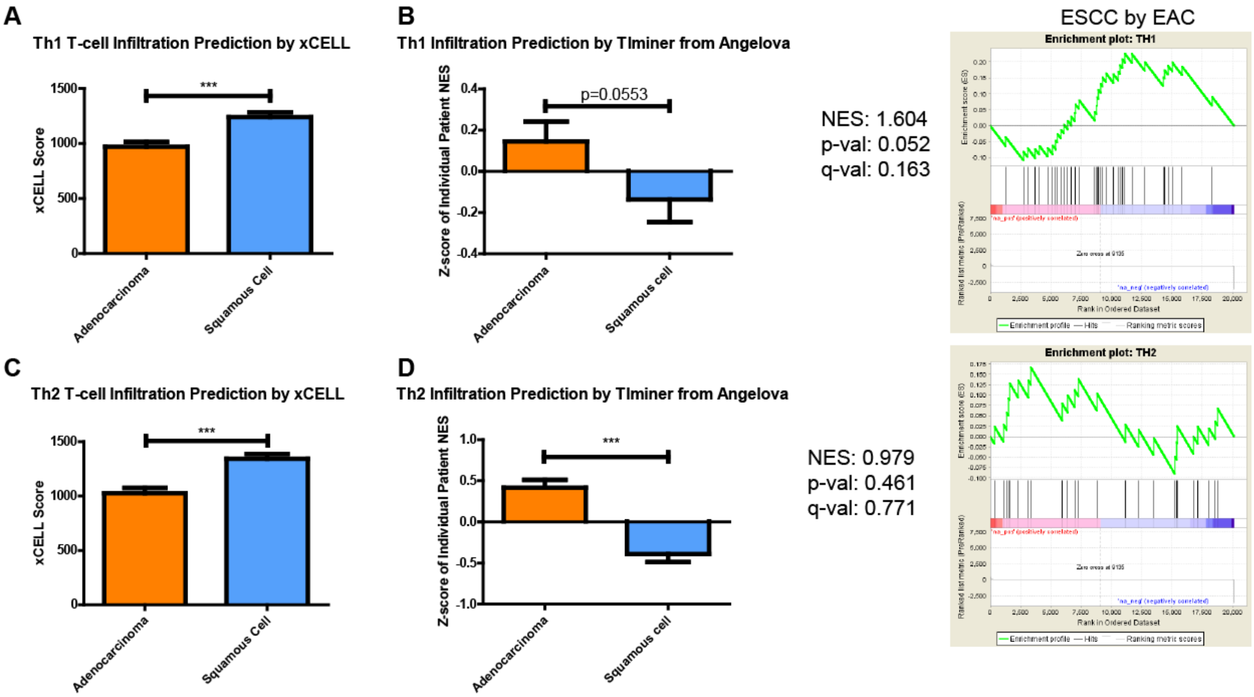

Supplementary Figure 14. Helper T-cell prediction in histological subtypes. CD4 helper T-cell infiltration prediction from several algorithms. This includes (A) Th1 by xCell, (B) Angelova's signature for Th1 infiltration by TIminer, including individually calculated normalized enrichment scores (left) and group comparison between subtypes (right), (C) Th2 infiltration by xCell, (D) and Th2 infiltration by TIminer, including individually calculated Z-scores (left) and group comparison between subtypes (right). Gene set enrichment analysis (GSEA) was calculated by dividing the average group RSEM for ESCC by EAC's average. Statistics supplied by GSEA with q-value indicating Benjamini-Hochberg's correction. ***P < 0.001.

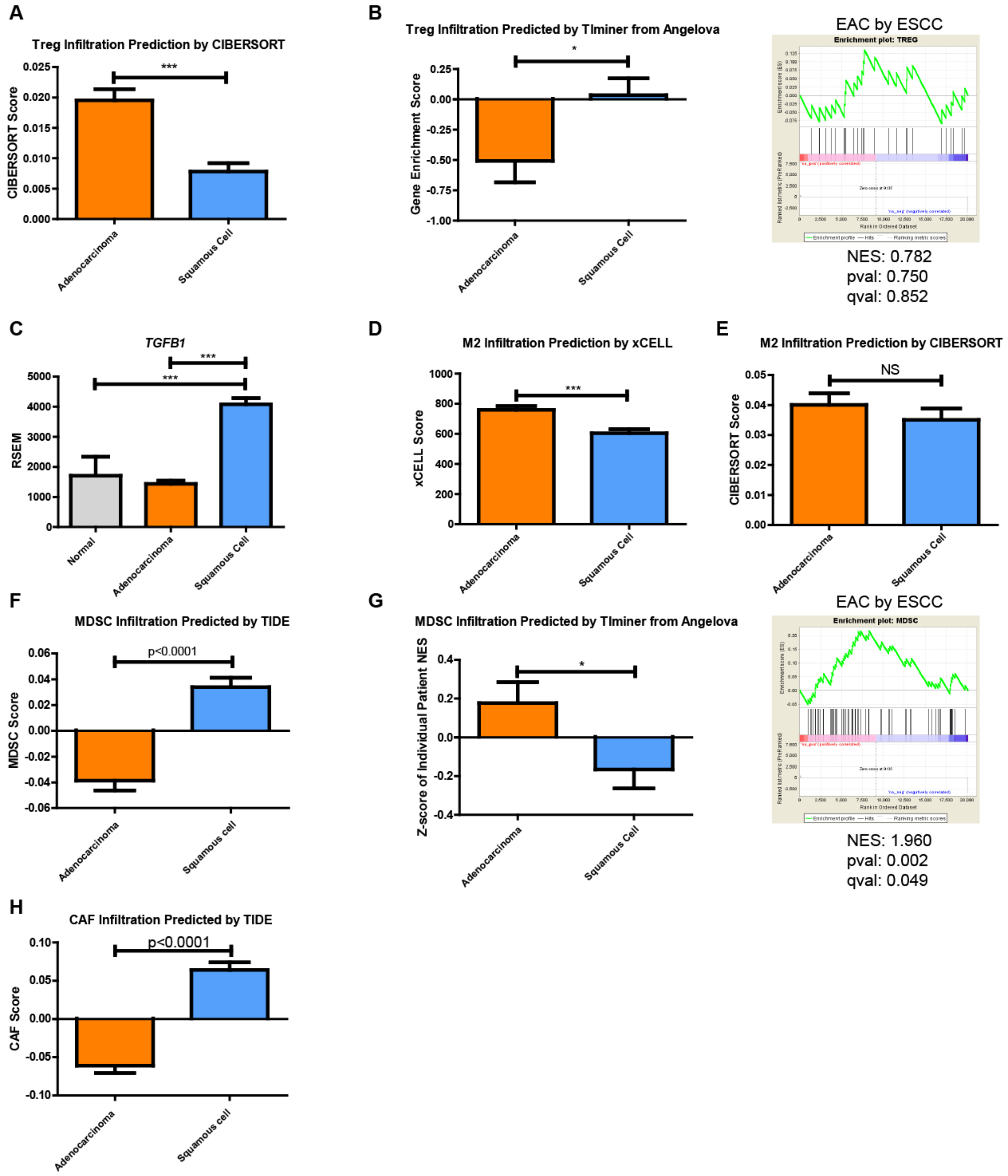

Supplementary Figure S15. Possible inhibitory infiltration prediction in histological subtypes. (A-H) Inhibitory anti-inflammatory immune infiltration was examined, but an obscure status was seen through several algorithms. This includes Treg infiltration by CIBERSORT (A), Angelova's signature for Treg infiltration by TIminer, including the normalized enrichment score of each patient compared to normal (left) and group comparison between subtypes (right) (B), mRNA expression of *TGFB1* (C), M2 infiltration predicted by xCell (D), M2 infiltration predicted by

CIBERSORT (E), MDSC infiltration predicted by TIDE (F), and with Angelova's signature through TIminer with individually calculated normalized enrichment scores (left) and group comparison between subtypes (right) (G). (H) Cancer-associated fibroblast (CAF) infiltration predicted by TIDE. Gene set enrichment analysis (GSEA) was calculated by dividing the average group RSEM for EAC by ESCC. Statistics supplied by GSEA with q-value indicating Benjamini-Hochberg's correction. A Student's t-test was conducted for significance for immune infiltration and a Kruskal–Wallis H test with Dunn's multiple comparison test between all columns for *TGFB1* expression. *P < 0.05, ***P < 0.001.

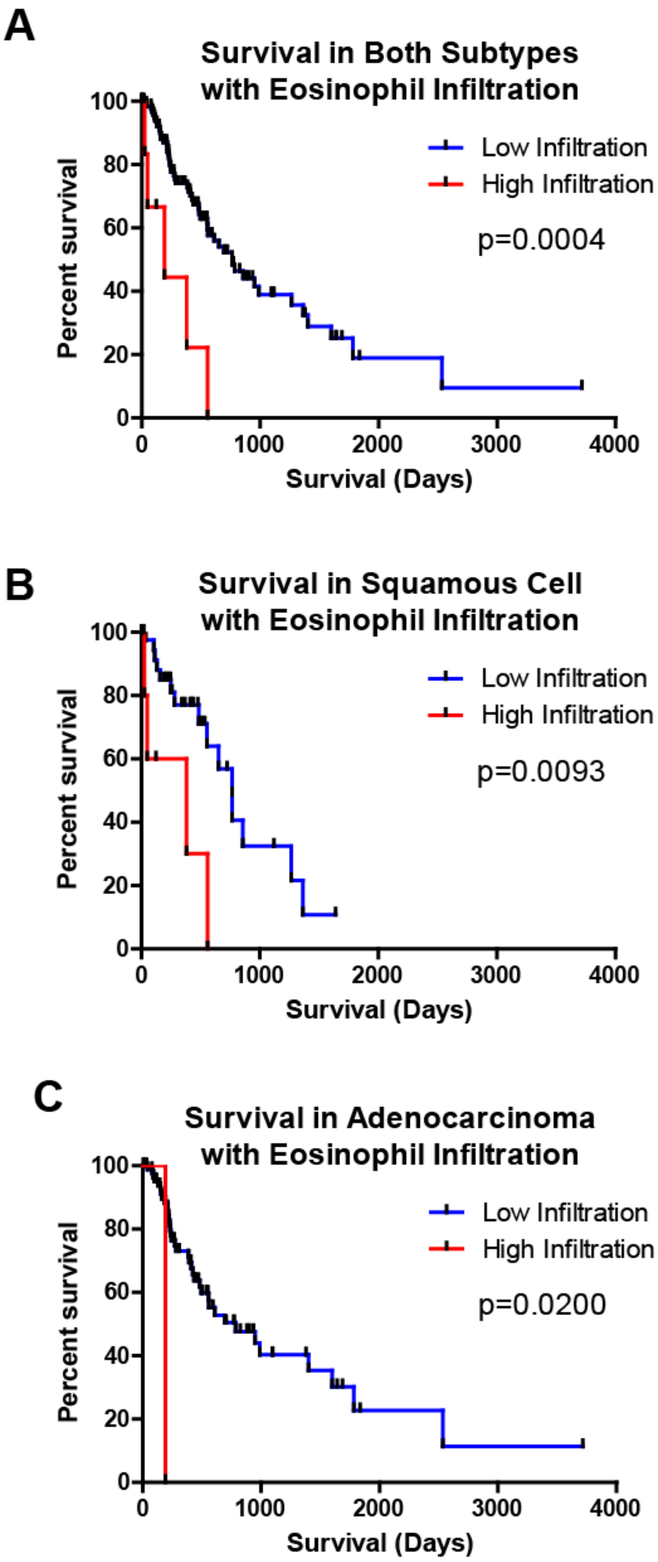

Supplementary Figure 16. Eosinophil infiltration impacts survival. Patient survival based on if patients had more or less than the median CIBERSORT score for eosinophil infiltration. Kaplan-Meier curves with Mantel-Cox log-rank test then examined (A) the entire esophageal cohort, (B) only ESCC, (C) and only EAC.

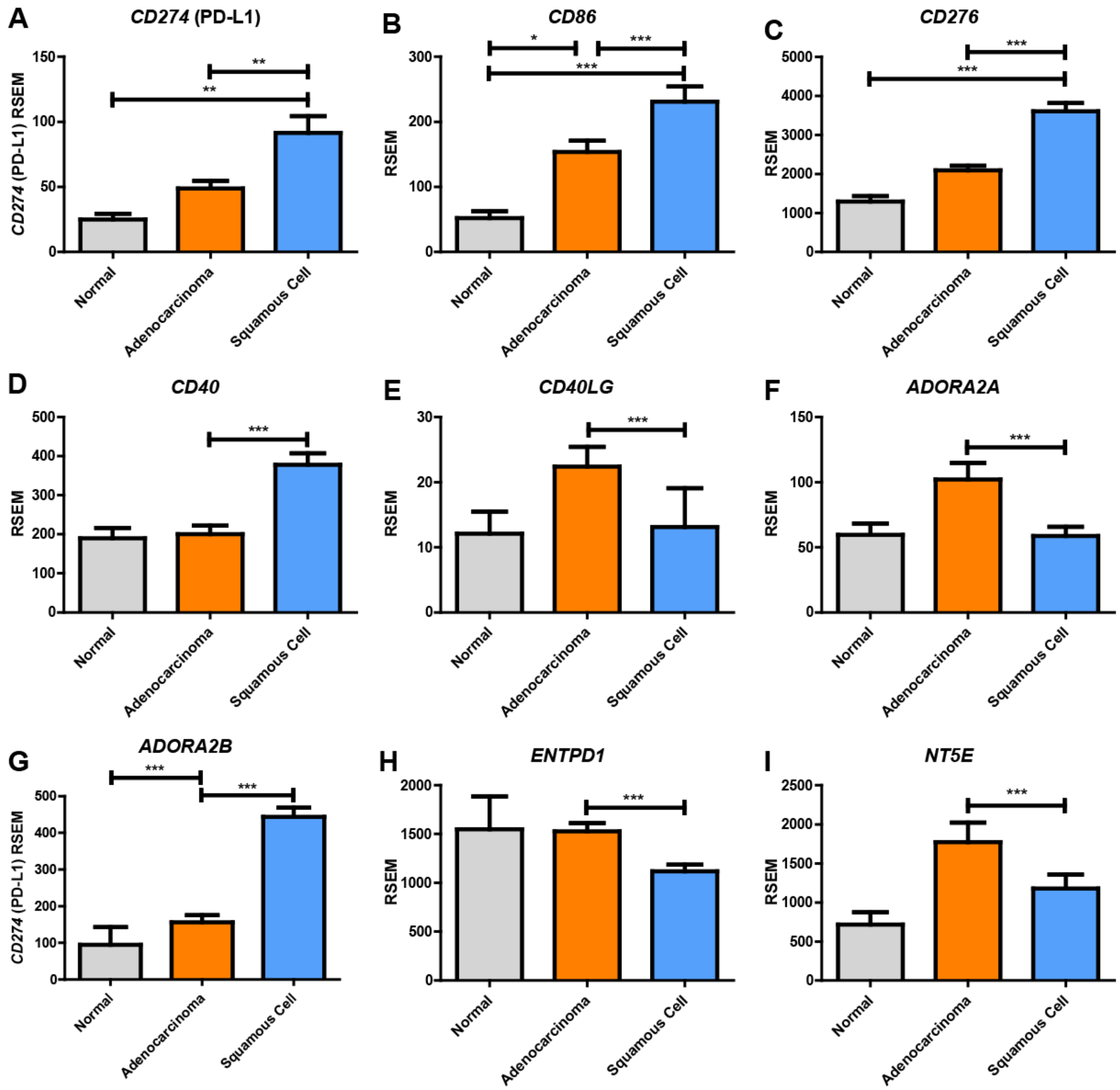

Supplementary Figure S17. mRNA expression of potentially targetable immune altering genes. (A-I) Potentially targetable prevalent genes associated with immune function and infiltration were examined for their mRNA expression. This includes *CD274* (A), *CD86* (B), *CD276* (C), *CD40* (D), *CD40LG* (E), *ADORA2A* (F), *ADORA2B* (G), *ENTPD1* (H), and *NT5E* (I). Statistical comparisons were made by using Kruskal–Wallis H test with Dunn's multiple comparison test between all columns. *P < 0.05, **P < 0.01, ***P < 0.001.

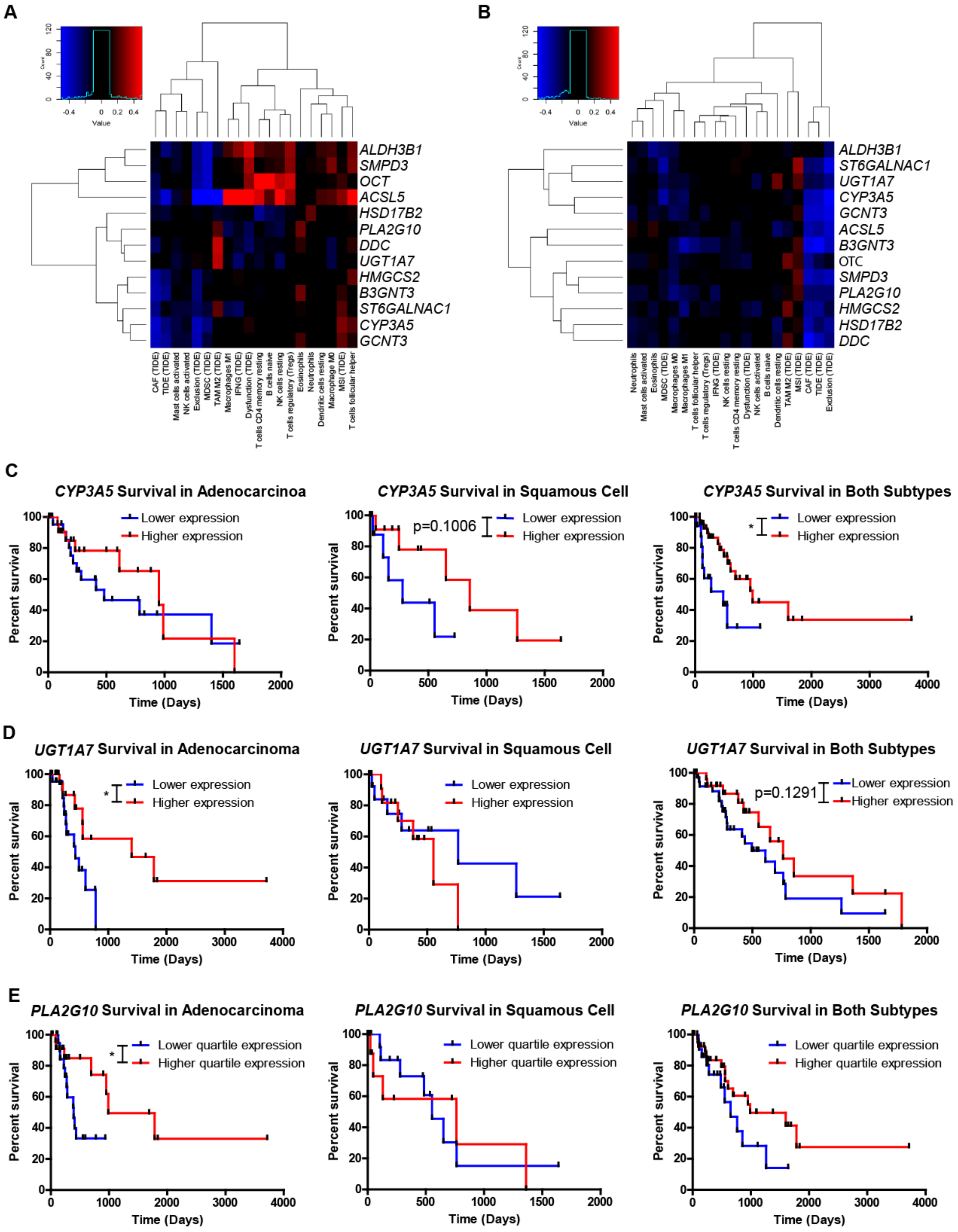

Supplementary Figure 18. Enzymes associated with immune alteration and their impact on survival. The correlation of significantly altered enzyme expression was further examined

between cohorts, along with the resulting impact on survival, to examine the impact of differences in histological subtypes. Heatmaps showing Spearman's rho were constructed to examine the 13 significant genes within (A) ESCC and (B) EAC utilizing CIBERSORT and TIDE. The 13 genes were examined for their impact on survival, but significance was only seen with (C) *CYP3A5*, (D) *UGT1A7*, (E) and *PLA2G10* for EAC (left), ESCC (middle), or both subtypes (right).

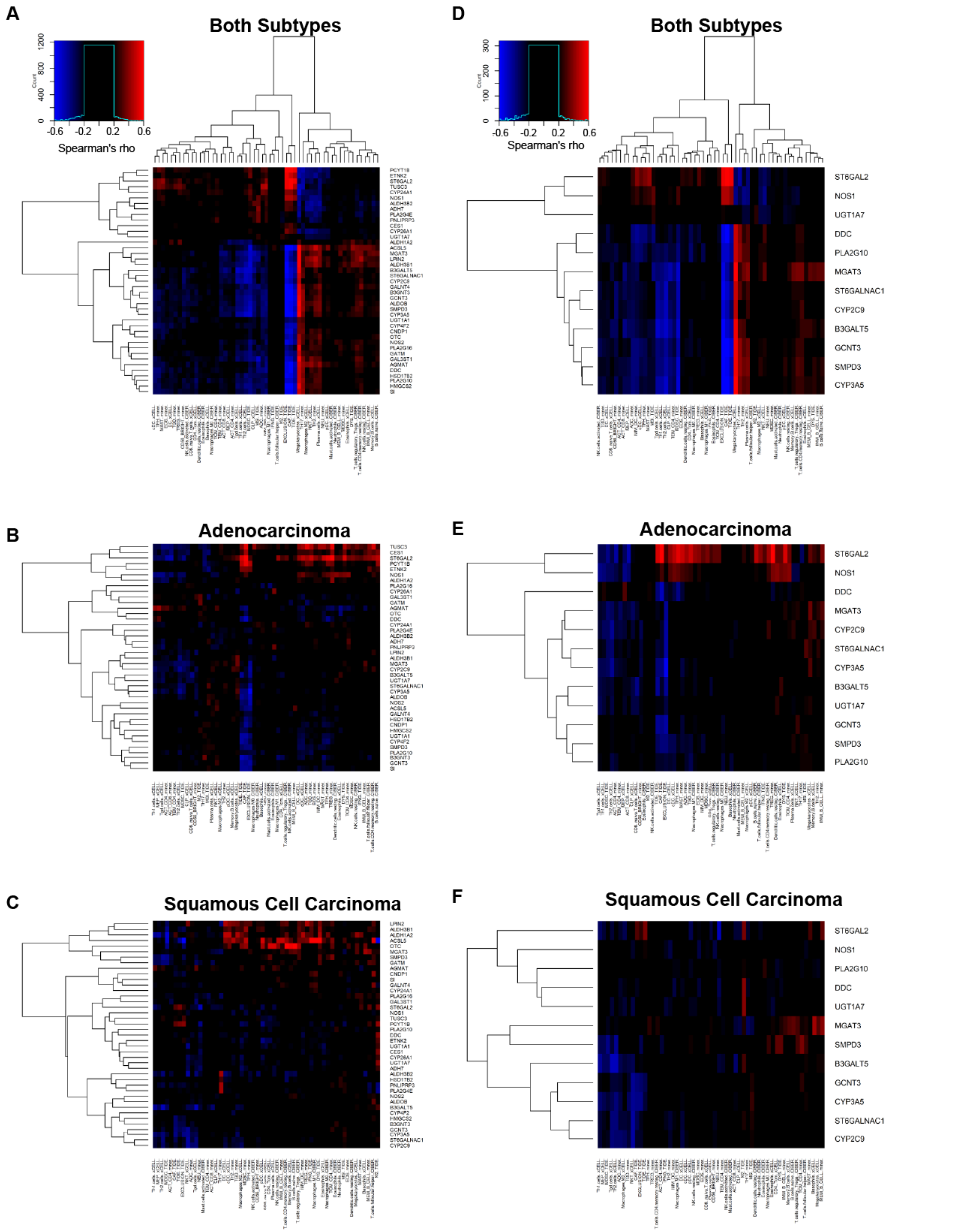

Supplementary Figure 19. Differentially expressed enzyme correlation with all algorithms for immune function. Immune correlation with enzymes was examined with CIBERSORT, xCell, TIminer with Angelova, and TIDE. Enzymes were correlated with immune predictions and then

plotted as heatmaps with Spearman's rho if the genes were contained within the top or bottom 100 Spearman rhos among enzymes, which were then filtered if they had a minimum of 5-fold change with $p < 0.05$ (Student's t-test) and impacted at least one category when comparing both subtypes. The resulting heatmaps were then plotted for (A) both subtypes, (B) adenocarcinomas, and (C) squamous cell carcinomas. To refine results to identify enzymes of the greatest significance, enzymes were plotted when they were found to play a significant role in at least 5 categories in both subtypes for (D) all esophageal patients, (E) adenocarcinomas, and (F) squamous cell carcinomas.

Supplementary Table Legends:

Supplementary Table 1. Survival impact of enzymes between histological subtypes. A Mantel-Haenszel log-rank test was conducted for all enzymes between patients containing the top or bottom 25% mRNA expression within each histological subtype or when combined for the entire esophageal cohort.

Supplementary Table 2. Survival impact of all genes between histological subtypes. A Mantel-Haenszel log-rank test was conducted for all genes between patients containing the top or bottom 25% mRNA expression within each histological subtype or when combined as the whole esophageal cohort.

Supplementary Table 3. Gene ontology biological process enrichment scores for esophageal patients. A summary is provided for the gene set enrichment analysis conducted for all esophageal patients, which was completed by comparing each patient individually to the average of the adjacent normal tissue. Data were then examined for significance utilizing a Student's t-test and a Mann-Whitney U test, with q-values being derived from the Benjamini-Hochberg (BH) correction. Fold change is defined as a ratio, or the negative inverse when the ratio was less than one. The last column indicates the absolute value of the fold change of the squamous histology divided by the adenocarcinoma histology.

Supplementary Table 4. KEGG enrichment scores for all esophageal patients. A summary is provided for the gene set enrichment analysis conducted for all esophageal patients, which was completed by comparing each individually to the average of the adjacent normal tissue. Data were then examined for significance utilizing a Student's t-test and a Mann-Whitney U test, with q-values being derived from the Benjamini-Hochberg (BH) correction.

Supplementary Table 5. mRNA expression of genes with gene ontology term cytokine activity. Average mRNA expression from TCGA was collected into histological subtypes or adjacent normal tissue for genes contained within gene ontology term "cytokine activity" (GO: 0005125).

Supplementary Table 6. Biomarker differences between subtypes and normal tissue. Each available gene in the TCGA was examined for potential differences between each subtype and normal tissue. Table shows fold change (FC), Student's t-test p-value (pval), and area under the curve (AUC). The far right three columns represents the average of the group. When comparing FC, the cohort on the left is the numerator and the right side is the denominator. Absolute FC indicates the max relative FC absolute value, meaning the absolute value was taken of the denominator and numerator were switched if the denominator was larger. For genes without

expression in one of the cohorts, “NA” and “Inf” are given with a potential early row termination, as further examination yields little-to-no benefit.
